# Tracking the Pliensbachian–Toarcian Karoo firewalkers: Trackways of quadruped and biped dinosaurs and mammaliaforms

**DOI:** 10.1371/journal.pone.0226847

**Published:** 2020-01-29

**Authors:** Emese M. Bordy, Akhil Rampersadh, Miengah Abrahams, Martin G. Lockley, Howard V. Head

**Affiliations:** 1 Department of Geological Sciences, University of Cape Town, Cape Town, South Africa; 2 Dinosaur Tracks Museum, University of Colorado Denver, Denver, Colorado, United States of America; Southern Illinois University, UNITED STATES

## Abstract

The Karoo igneous rocks represent one of the largest continental flood basalt events (by volume) on Earth, and are not normally associated with fossils remains. However, these Pliensbachian–Toarcian lava flows contain sandstone interbeds that are particularly common in the lower part of the volcanic succession and are occasionally fossiliferous. On a sandstone interbed in the northern main Karoo Basin, we discovered twenty-five tridactyl and tetradactyl vertebrate tracks comprising five trackways. The tracks are preserved among desiccation cracks and low-amplitude, asymmetrical ripple marks, implying deposition in low energy, shallow, ephemeral water currents. Based on footprint lengths of 2–14 cm and trackway patterns, the trackmakers were both bipedal and quadrupedal animals of assorted sizes with walking and running gaits. We describe the larger tridactyl tracks as “grallatorid” and attribute them to bipedal theropod dinosaurs, like *Coelophysis*, a genus common in the Early Jurassic of southern Africa. The smallest tracks are tentatively interpreted as *Brasilichnium*-like tracks, which are linked to synapsid trackmakers, a common attribution of similar tracks from the Lower to Middle Jurassic record of southern and southwestern Gondwana. The trackway of an intermediate-sized quadruped reveals strong similarities in morphometric parameters to a post-Karoo Zimbabwean trackway from Chewore. These trackways are classified here as a new ichnogenus attributable to small ornithischian dinosaurs as yet without a body fossil record in southern Africa. These tracks not only suggest that dinosaurs and therapsids survived the onset of the Drakensberg volcanism, but also that theropods, ornithischians and synapsids were among the last vertebrates that inhabited the main Karoo Basin some 183 Ma ago. Although these vertebrates survived the first Karoo volcanic eruptions, their rapidly dwindling habitat was turned into a land of fire as it was covered by the outpouring lavas during one of the most dramatic geological episodes in southern Africa.

## Introduction

**Fig 1:**
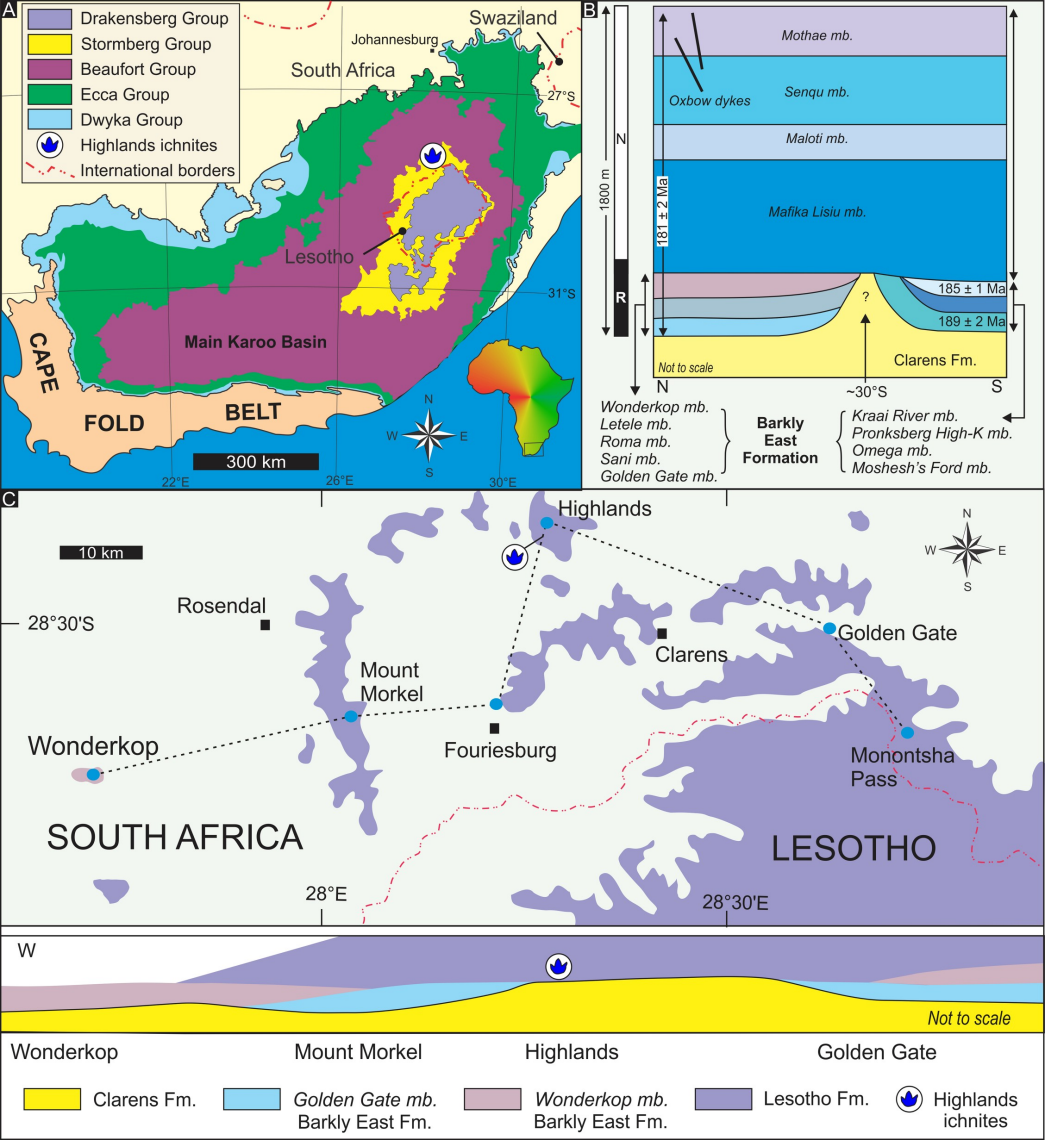
Location and stratigraphy of the Highlands region in the main Karoo Basin of South Africa. A. Simplified geological map of the main Karoo Basin (modified from [9]). B. Generalized stratigraphic column of the Drakensberg Group (modified from [10] with dates from [11], p. 758, 762). C. Simplified geological map and cross-section of the Highlands region in the eastern Free State of South Africa (modified from [12]). Figs A, B and C modified and republished from [9, 10, 12] under a CC BY license, with permission from the Council for Geoscience and Geological Society of South Africa, original copyright 2008, 2009 and 1984, respectively.

The main Karoo Basin of southern Africa (Fig 1) is an excellent study area for land-based manifestations of several Palaeozoic and Mesozoic mass extinction events. For example, the end-Triassic event, which occurred during the deposition of the Elliot Formation (Stormberg Group), marks a global faunal turnover event that is generally considered as the third largest of five major biological crises in the geological record [1–3]. While the end-Triassic event is firmly linked to the outpouring of the continental flood basalts of the Central Atlantic Magmatic Province, the next mass extinction event at the end-Pliensbachian (Pliensbachian-Toarcian extinction) is related to the volcanic events of the Karoo-Ferrar Large Igneous Province (e.g., [4–8]). Being one of the largest igneous provinces on Earth, the Karoo-Ferrar Large Igneous Province extended from the Karoo, across Antarctica to South Australia with a total length of > 5000 km in the Early Jurassic, and its rock record is best exposed in the main Karoo Basin.

Associated with these large igneous events, major possible environmental perturbations for life were the increase in atmospheric CO_2_ and other gases from volcanic degassing and wildfires causing warming, volcanic dust causing dimming of daylight, volcanic winters, food chain collapse, sea level fluctuations, methane hydrate release and perturbations in the global biogeochemical cycles leading to oceanic anoxia (e.g., [11, 13–31]). During mass extinctions, these external events filtered the major groups of organisms, and allowed, for example dinosaurian and mammalian ecological dominance after end-Triassic and end-Cretaceous events, respectively. However, in mass extinction research, the role of environmental filters and key physiological innovations in continental communities are poorly understood. Furthermore, while Jurassic and Cretaceous continental biomes also contained mammals (first recorded from the upper Elliot Formation globally), the environmental conditions for dinosaur-mammal co-existence have not yet received a satisfactory explanation. Finally, the uncertainty in determining the controlling factors and their relative contributions to the Toarcian biotic turnover on land is ultimately associated with: (a) the low number of complete and well-dated Lower Jurassic continental sections, (b) difficulties in correlating the results of the different stratigraphic studies (e.g., biostratigraphy, isotope stratigraphy, magnetostratigraphy) on globally dispersed sections, and (c) lack of modern equivalents for massive continental scale volcanic events (e.g., [31, 32]).

Notwithstanding the above challenges and the incomplete global rock record from this period, the vast upper Karoo rock exposures suggest that the main Karoo Basin has a supreme data archive for interrogating the impact of the above potential environmental forcing mechanisms on the Early Jurassic continental biomes of Gondwana. The upper Karoo Supergroup of southern Africa, encompassing the upper Stormberg and lower Drakensberg Groups, is one of the few stratigraphic units globally that contains diverse continental rocks as well as body and trace fossil assemblages to allow a meaningful assessment of the events leading up to the Toarcian turnover, a global multi-phased extinction event. This area is also unique because it is most proximal to the trigger of the event: the Karoo-Ferrar flood basalt outpourings (e.g., [11, 12, 33, 34]), and preserves the rock record of this globally significant event in spectacular outcrops suitable for multidisciplinary investigations.

Although the fossil heritage of the lower Drakensberg Group is not comparable to that of the lowermost Jurassic units in the upper Karoo (upper Elliot and Clarens formations–see [35–37] for reviews), this Pliensbachian-Toarcian unit still preserves important and diverse remains of past life. Here we present high resolution ichnological and sedimentological findings from a sandstone interbed in the basalts of the Drakensberg Group and characterize the palaeoclimate and palaeoenvironment of southern Africa at the turn of the Pliensbachian-Toarcian. We describe a new ichnotaxon of ornithischian affinity together with additional ichnites of mammaliaform and theropod affinities in their original geological context, increasing the ichnodiversity of this volcano-sedimentary unit. Moreover, indirectly our results help refine the relationship between the continental ecosystems in the main Karoo Basin to the Pliensbachian-Toarcian global biotic crisis event, which originated in the Karoo-Ferrar region.

## Geological background

### Stratigraphy of the lower Drakensberg Group

The fossiliferous upper Karoo (upper Elliot and Clarens Formations and lower Drakensberg Group) is present throughout southern Africa (e.g., [38–42]), and has therefore remarkable significance for evaluating not only the Early Jurassic evolutionary changes in southern Gondwana, but also the environmental changes leading up to the end-Pliensbachian global event. In recent years, regional-scale sedimentary facies analysis and palaeoenvironmental reconstruction of the upper Karoo in southern Africa showed that the upper Elliot Formation (Hettangian-Sinemurian) resulted from semi-arid, ephemeral fluvial and lacustrine processes [39, 40, 43]. Furthermore, it has been long established that the fine- to medium-grained sandstones of the Clarens Formation (Sinemurian-Pliensbachian) were predominantly deposited in a wet to dry desert environment with dominant easterly palaeo-winds before the outpouring of the continental flood basalts of the Karoo-Ferrar Large Igneous Province at 183±1 Ma (e.g., [42, 44–47]).

The continental flood basalt succession, the Pliensbachian-Toarcian Drakensberg Group (Fig 1), is subdivided into the lower Barkly East Formation (~ 300 m thick) and the upper Lesotho Formation (~ 1300 m thick; e.g., [11, 12, 33, 48, 49]). The former comprises igneous rocks (tholeiitic basalt, pillow lavas, pyroclastics, andesitic breccia, andesite, agglomerate) with heterogeneous geochemical composition and spatial distribution as well as fossiliferous sandstone and rare mudstone interbeds that range in thickness from a few 10s of cm to several metres. Comparatively, the basaltic lava flows of the Lesotho Formation contain fewer sedimentary interbeds, and interflow weathering features, which indicate, among other lines of evidence (e.g., [11, 12, 33, 48, 49]), a rapid outpouring of the main part of the lavas, which may have been taken place over a period as short as 250 ka (e.g., [11, 33, 49]). Furthermore, this also suggests that during the main phase of lava eruptions the clastic sediment supply to the main Karoo Basin was limited and recycling of the older Karoo strata was negligible mostly likely due to the rapidly expanding continental flood basalt blanket that was sprawling over the entire southern African region (e.g., [33, 48, 49]).

Both the magnetostratigraphy and chemostratigraphy of the lava pile (Fig 1) have been well-established, and recently there have been major advances in determining the time frame of its eruptive history (e.g., [11, 33, 49]). In northern Lesotho (Fig 1), the radiometric age of the Barkly East Formation ranges from 180.1 ± 2.2 to 182.8 ± 2.6 Ma, being essentially coeval with the main phase of Karoo volcanism (i.e., the Lesotho Formation) with a peak age of 183 ± 1 Ma and age range of 181–183 Ma [6, 11, 46, 50]. In the south, the lowermost basalts appear to be ~ 6 Ma older (^40^Ar/^39^Ar plateau age: 189 ± 2 Ma—[11]) as suggested by not only absolute dating methods but also cross-cutting field relationships [11, 51]. This geochronological framework, although not based exclusively on high precision dates, supports not only a multiphased eruption history, and a very short time span for the main lava pile emplacement, but also the pulsating nature of the global environmental and biotic perturbations during the Pliensbachian-Toarcian interval.

It has been shown (e.g., [33, 48]) that regionally the top of the Clarens Formation is an irregular albeit a low relief palaeosurface onto which the first lava flows were emplaced. In the Highlands region (Fig 1), the thickness of the Clarens Formation ranges from 60 to 140 m over a distance of 3–4 km, and the maximum preserved thickness of the lower Drakensberg Group is ~ 125 m. The contact between the Clarens sandstones and the Karoo basalts is easily mappable, and as documented by Kingsley [52], it is a surface with a relatively smooth topography with a relief of only a few 10s of metres over a few kilometres. Furthermore, Marsh [12], using field mapping and basalt chemostratigraphy, has shown that the same contact forms a domal structure around Highlands (Fig 1) and that the lowermost basalt flows here are part of the Lesotho Formation. By implication, the sandstone interbeds in the lava succession on Highlands, ~ 45 m above the upper contact of the Clarens Formation, are also part of the lowermost Lesotho Formation, and thus the Highlands trace fossils were probably generated around the Pliensbachian–Toarcian transition, some 183 Ma ago. Generally, in the main Karoo Basin, the sedimentary interbeds are more persistent and abundant in the Barkly East Formation than in the Lesotho Formation (e.g., [11]), and consist of sheets and lenses of sandstones and very rare mudstones of limited lateral extent. The thickness of the interbeds ranges from a few centimetres to several tens-of-metres (e.g., maximum reported thickness in the southern main Karoo Basin is 60 m), however typically they are 2–3 m thick (e.g., [33, 48, 52]. The sandstones are normally well-bedded, clayey to silty very fine- to medium-grained, quartz-rich, and contain horizontal lamination, cross-bedding, ripple cross-lamination, desiccation cracks and a moderately diverse fossil assemblage (see next section). The relative abundance of pillow lavas and lack of sedimentological evidence for definite aeolian origin in the interbeds imply a palaeoenvironment with sheet floods, temporary streams, very rare dunes, and scattered ephemeral ponds or lakes into which some detrital sediments accumulated, and earliest lavas flowed.

### Palaeontology of the lower Drakensberg Group

The fossils in the Pliensbachian-Toarcian sedimentary interbeds range from vascular plants including arthrophytes, conifers, cycads, equisetites, petrified gymnosperm wood with well-developed growth rings, charred tree trunks within *in situ* forests, plant roots/rootlets, a variety of arthropods including malacostracans, conchostracans, notostracans, tiny vertebrates of unknown affinity (5–7 mm long bones, ?pelvic element), potential fish remains, invertebrate trails and tetrapod trackways (e.g., [44, 52–54]). Remarkably, tracksites in the Drakensberg Group of central Lesotho (e.g., Ralikhomo, Lekhalo-la-maburu) that are stratigraphically ~ 450 to ~ 700 m above the Highlands ichnosite, well within the continental flood basalt pile, preserve mostly small, tetradactyl tracks. These tracks have been attributed to mammalians that were bipeds and quadrupeds with marked heteropody (some habitual hoppers), and were assigned to the ichnogenus *Malutitetrapodiscus* (e.g., *M*. *saltator*, *M*. *tenuis*, *M*. *minimus*, *M*. *perlinax*–e.g., [54–58]). To date, the only exception to the strong mamaliaform affinity of the central Lesotho tracks is one tridactyl track, *Ralikhomopus aviator*, with imprints of feather-like structures around the toes, markings that were later re-assessed to be invertebrate trails ([59], p. 60). *Ralikhomopus aviator* was attributed to a small, bird-like dinosaurs by Ellenberger [56], which he called “tiny proto-avian of Drakensberg".

## Materials and methods

The trace fossil assemblage at the Highlands ichnosite (geographical coordinates: 28°24'53.50"S 28°15'11.14"E) was studied both in the field and laboratory after obtaining their photogrammetric models and silicon rubber replicas, by: describing their architectural and surficial morphologies, obtaining their physical and digital morphometric measures for in-depth descriptions and for ichnotaxonomic treatment. Standard ichnological methods as outlined in MacEachern et al. [60, 61]; Falkingham [62]; Lallensack et al. [63]; Sciscio et al. [64] and Falkingham et al. [65] were followed. In addition, vertical and regional changes in the host sedimentary facies architecture were described in the field by applying principles of facies analysis (e.g., measurement of grain size, thickness, palaeocurrents, sedimentary structures) following standard sedimentological methods as outlined in Miall [66–69]. Relevant outcrops and their sedimentary and volcanic features were captured in the field in the form of photomosaics, sketches and geological field mapping. Orthogonal photographs of individual tracks, trackways and the spatial concentration of the footprints were captured on site using a Canon PowerShot EOS D1200 (Focal length 28 mm, 5184 x 3456 resolution). Additional close-range photographs of the track-bearing surface were taken in the field in order to reconstruct 3D photogrammetric models of the best-preserved tracks and the palaeosurface. The models were generated using Agisoft Photoscan software (standard version 1.1.4) following the procedures of Mallison and Wings [70]. Depth-colour maps of individual tracks and trackways were created using Cloud Compare (software v.2.6.1). Ichnological photogrammetric data including field photographs used in the photogrammetric models, and the cleaned and aligned 3D models are available here: **https://doi.org/10.6084/m9.figshare.7442468**. Contour lines for some individual tracks were created using ParaView (software v.5.2.0). Silicon rubber casts of trackways A and B (BP/6/744 and BP/6/745, respectively) are housed in the Evolutionary Science Institute at the University of the Witwatersrand (Johannesburg, South Africa). This geoscientific study did not involve any endangered or protected species or locations, and did not require a specialist research permit because we did not remove any fossils or other samples from the field, however, because the study site is privately owned, we did obtain full permission from the landowner to work at the site.

**Fig 2:**
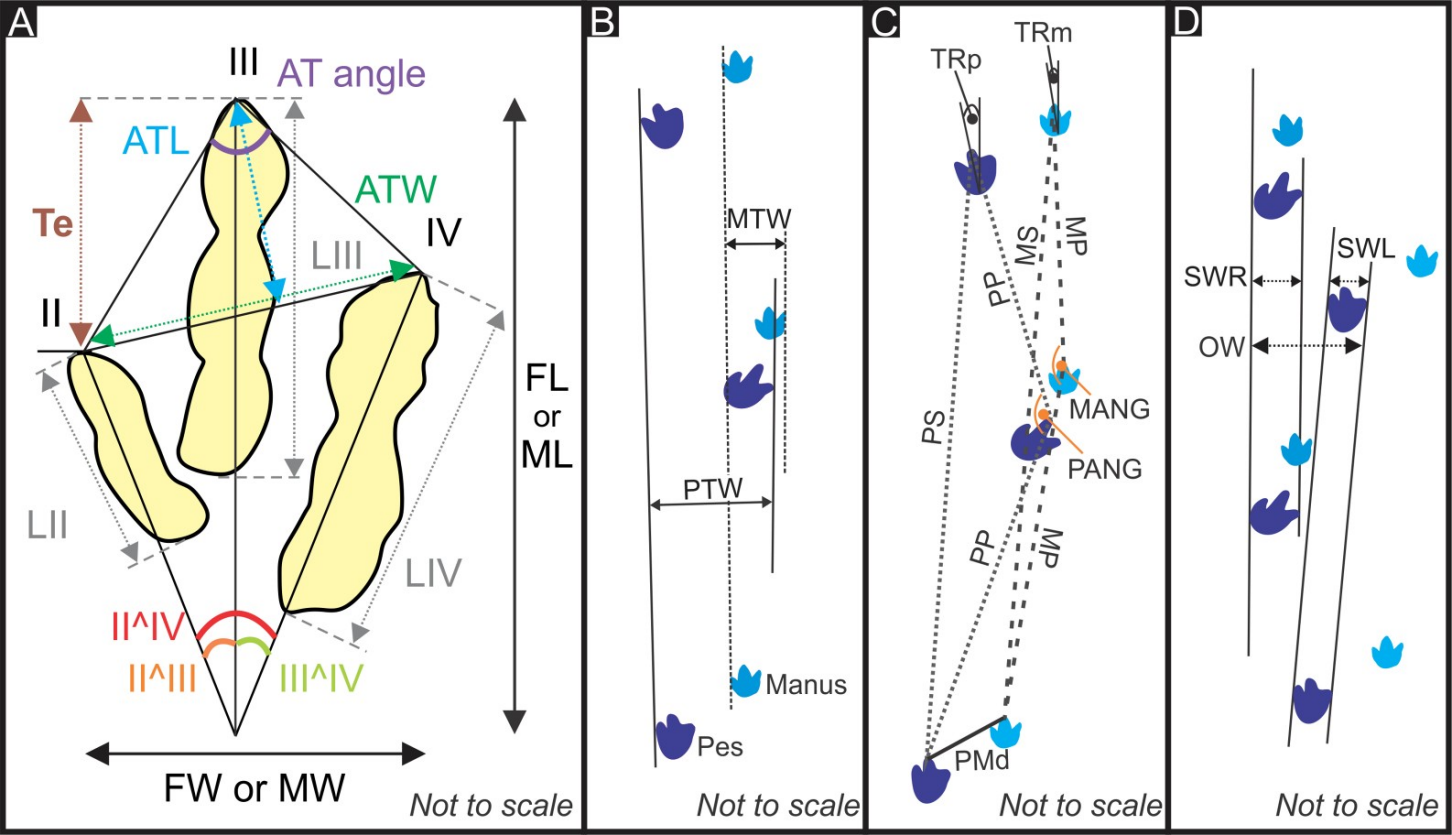
Schematic diagram showing the various track and trackway parameters measured. (A) Measurements taken on individual footprints. (B) Manus and pes trackway widths (MTW, PTW). (C) Trackway measurements. (D) Parameters used to quantify trackway gauge recommended by Romano et al. [71]. See methods text for a full list of abbreviations used.

**Fig 3:**
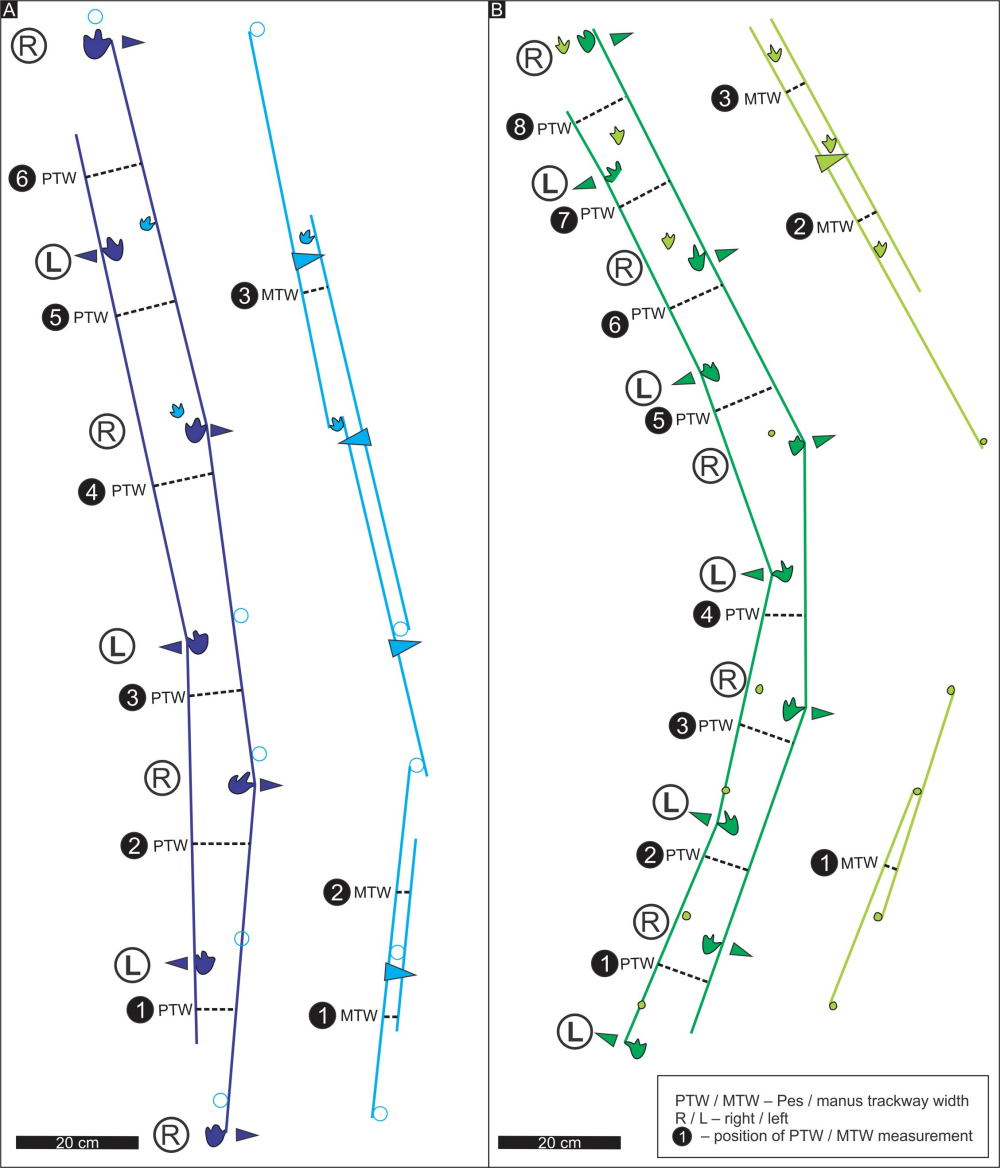
Position of the width measurements taken from the pes and manus trackways. (A) Highlands trackway A. (B) Chewore trackway. Chewore trackway modified and republished from Lingham-Soliar and Broderick [74] under a CC BY license, with permission from Taylor & Francis, original copyright 2000. Triangular arrows point away from the trackway midline.

Standard ichnological measurements of the tracks (Fig 2) were taken after Thulborn [59], Romano et al. [71], Castanera et al. [72] and Sciscio et al. [73]. The following parameters were measured, where applicable: pes length (FL), pes width (FW), anterior triangle length (ATL), anterior triangle width (ATW), manus length (ML), manus width (MW), pes pace (PP), pes stride (PS), manus pace (MP), manus stride (MS), pes to manus distance in each step (P-M distance), pes pace angulation (P ANG), manus pace angulation (M ANG), length of respective digits (LII, LIII, LIV), pes track rotation (TRp), manus track rotation (TRm), digit divarication (II^IV, II^III, III^IV), pes trackway width (PTW), manus trackway width (MTW) and pes trackway ratio (PTR). Measurements that were unobtainable in the field, such as digit divarication, were measured using photographs on ImageJ software. Measurements from Lingham-Soliar and Broderick [74] were incorporated into the dataset for comparative analysis and were measured using published track and trackway outlines (Fig 3). The measurements are summarized in the in-text tables and detailed in S1 Table. Trackmaker hip height (H) was calculated using the ratio for small theropods and ornithopods according to Thulborn [59]. The trackmaker’s gait was calculated using the relative stride length (λ/H), following Thulborn and Wade [75], which is expressed as the ratio of the average footprint stride length (λ) to hip height (H). Animals display a “walking” gait if λ/H ≤ 2.0, a “trotting” gait if 2.0 < λ/H < 2.9 and “running” gait for λ/H ≥ 2.9. Average locomotion speeds for trackmakers were estimated using the formula of Thulborn and Wade [75] for “running” gaits, and a combination of formulae from Alexander [76] and Thulborn and Wade [75] for “trotting” gaits.

## Results

### Stratigraphic and sedimentological context

**Fig 4:**
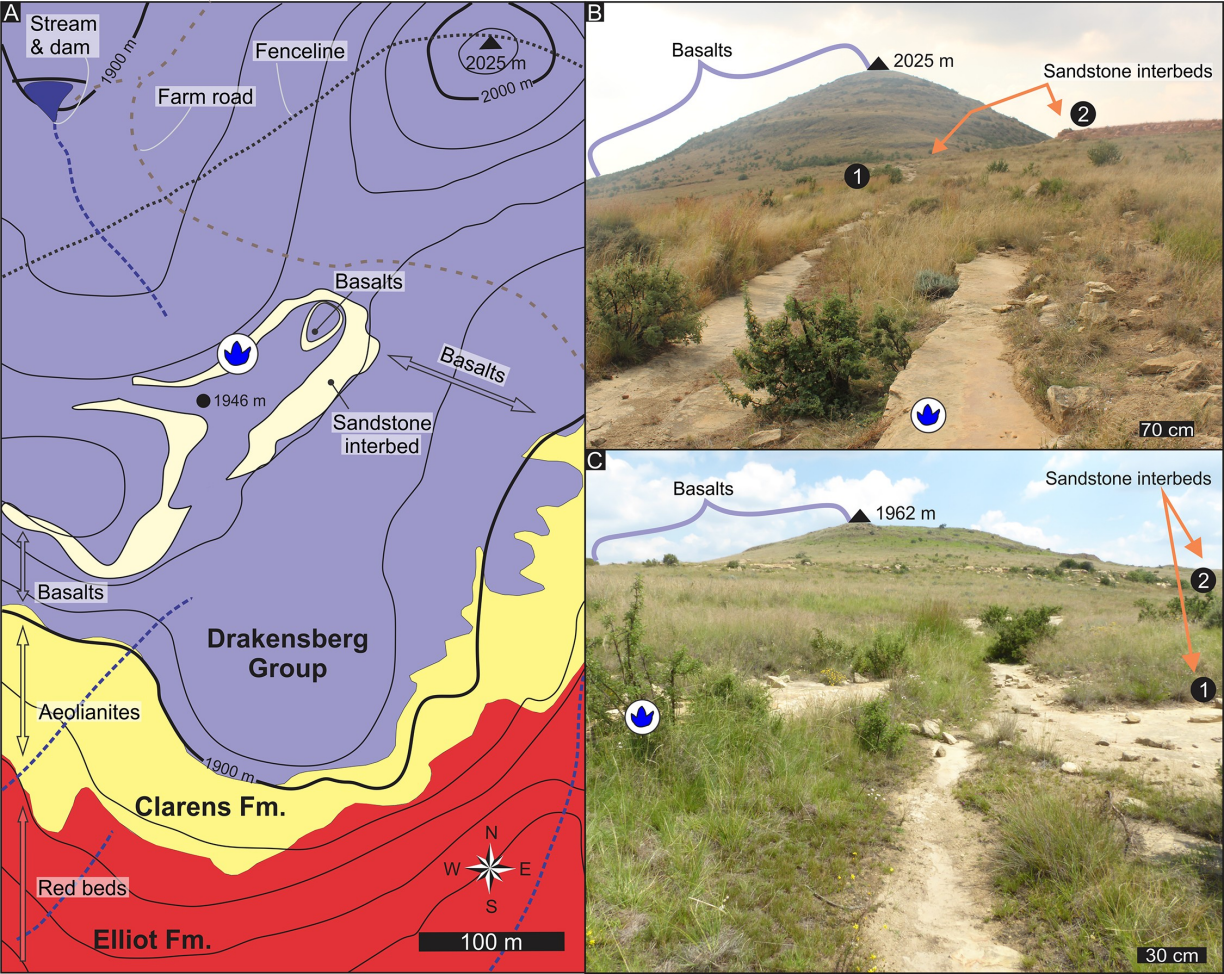
Geological context of the Highlands ichnosite. (A) Geological map of the vicinity of the Highlands ichnosite. (B) View of the interbed units and basalts to the NE and (C) to the SW from the track-bearing surface. Base map in A is a portion of 2828AD topographic map of the Chief Directorate of the National Geospatial Information (South Africa). This base map was republished under a CC BY license, with permission to reproduce under the Government Printer’s Authorization #11806 of 10 December 2018, original copyright 2001.

**Fig 5:**
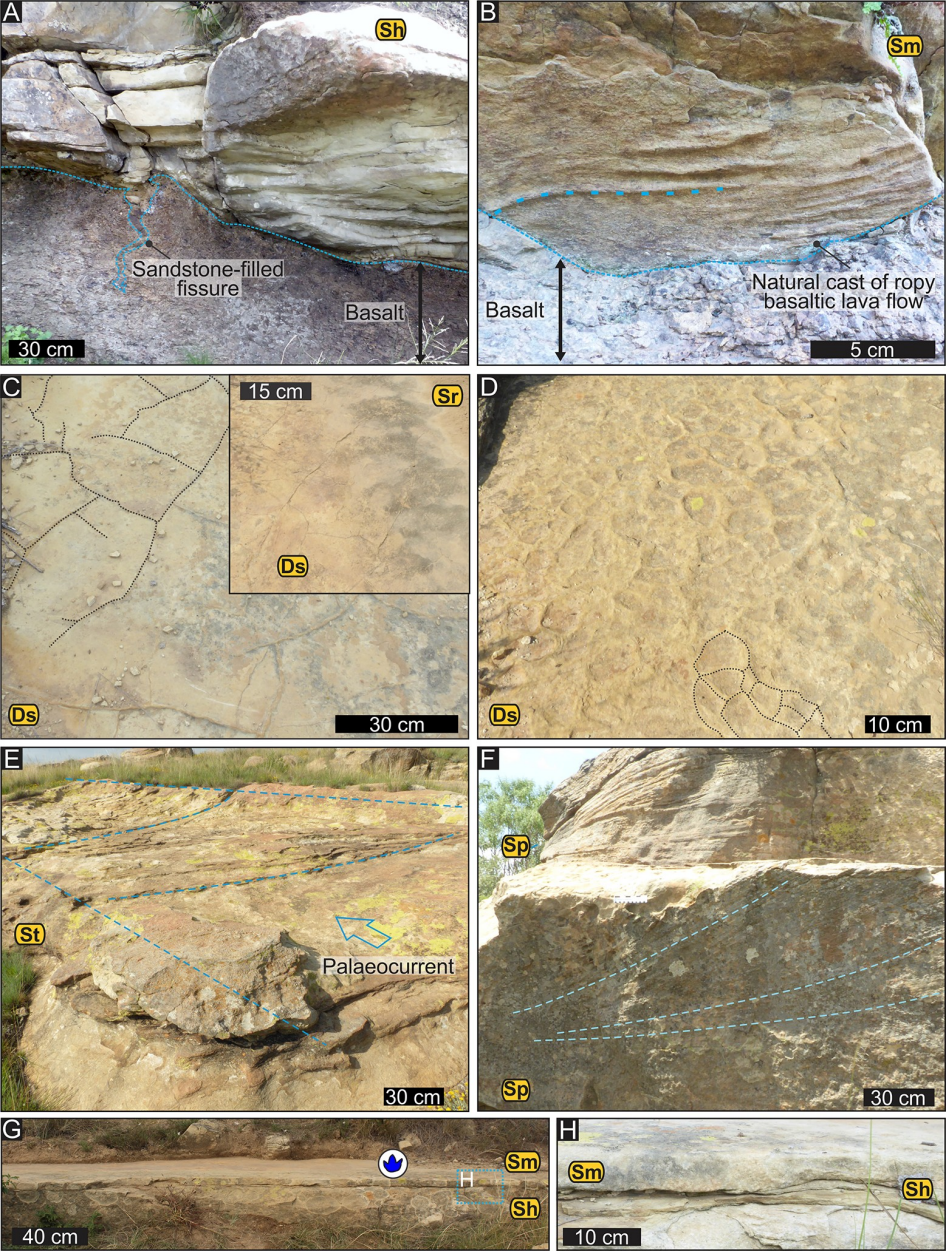
Sedimentary features associated with the sandstone interbeds on Highlands. (A) Horizontally laminated sandstone interbed unit overlying the basalt with a sandstone-filled fissure and a sharp but non-erosional contact. (B) Ropy-surface texture of pahoehoe lava flows preserved as a natural cast on the underside of a massive sandstone bed. These casts of the ropy-basalt surface suggest that the deposition of the sandstone interbeds occurred in non-erosive, and possibly rapid sedimentation events. (C) Casts of fine desiccation cracks and interference ripple marks (in the inset). (D) Casts of coarse desiccation cracks. (E) Large-scale trough cross-bedding in a 40-cm-thick medium-grained sandstone layer. (F) Planar cross-bedding with tangential, moderately inclined foresets in a ~ 120-cm-thick medium- to fine-grained sandstone layer. (G) Side-view of the track-bearing massive sandstone layer and underlying horizontally laminated sandstone bed. (H) Close-up of portion of G. Abbreviations: Sh–horizontally laminated sandstone; Sm–massive sandstone; Sr–ripple marks or ripple cross-laminated sandstone; St–trough cross-bedded sandstone; Sp–planar cross-bedded sandstone; Ds–desiccation cracks.

The Highlands ichnosite, containing an association of over 20 vertebrate tracks, is stratigraphically ~ 45 m above the upper contact of the Clarens Formation in the lower Lesotho Formation on Highlands 1239 farm (south of Bethlehem, eastern Free State, South Africa; Figs 1 and 4). The tracks are found on a single track-bearing surface that is the upper-bedding plane of a ~ 5-cm thick, massive, very fine to fine-grained sandstone layer, which is underlain by a horizontally laminated sandstone bed (Figs 4 and 5). The tracks, which are all on the same, essentially horizontal sandstone surface, are associated with horizontal, slightly meandering invertebrate trails and casts of desiccation cracks that range in width from a few mm to a few centimetres (Fig 5).

The two sandstone interbed units on Highlands farm are mappable for ~ 200 m horizontally along the hillslopes before they completely wedge out (Fig 4). The ichnosite is located within lower interbed unit (Fig 4B and 4C), which ranges in thickness from < 1 m SW of the ichnosite to ~ 15 m NE of the ichnosite. The sandstone interbed units are made up of lensoid to sheet-like sandstone layers with individual thicknesses that range from a few 10s of cm to ~ 1.5 m on Highlands. Very thin mudstone layers are also present within the interbed units, as attested by the casts of desiccation cracks, however their exposure is limited.

The lower contact of the sandstone interbeds seem to be sharp but non-erosional (Fig 5A; i.e., no evidence for incision). Instead, locally, along the sandstone-basalt contact, the underside of a sandstone bed preserves a remarkable ropy-structure typical on the upper surface of pahoehoe basaltic lava flows (Fig 5B). Well-developed, sandstone-filled fissures that penetrate from 0.3 to 0.7 m into the underlying basalt are locally common (Fig 5A) and have clean, sharp boundaries without chilled margins or veining. The upper contact of the sandstone interbeds with the overlying lava flows and high-quality exposures of the basalts have not been detected on Highlands due to the thick, grassy vegetation that typically develops over the basalts.

The sandstone interbeds consist of silty very fine- to medium-grained quartz-rich sandstones that are either massive, horizontally laminated, ripple cross-laminated, planar or trough cross-bedded (Fig 5C–5H). Vertical or lateral grain size changes are not apparent. The multiple sandstone surfaces preserve casts of desiccation cracks, ripple marks, and one surface contains traces of invertebrates and vertebrates (Figs 4 and 5C and 5D). Low-amplitude interference ripple marks and casts of desiccation cracks are exposed on the upper bedding plane of a sandstone layer that is immediately underlying the track-bearing sandstone layer (inset in Fig 5C), with smaller ripple marks of different orientation amongst the larger ripple marks.

### Ichnology

**Fig 6:**
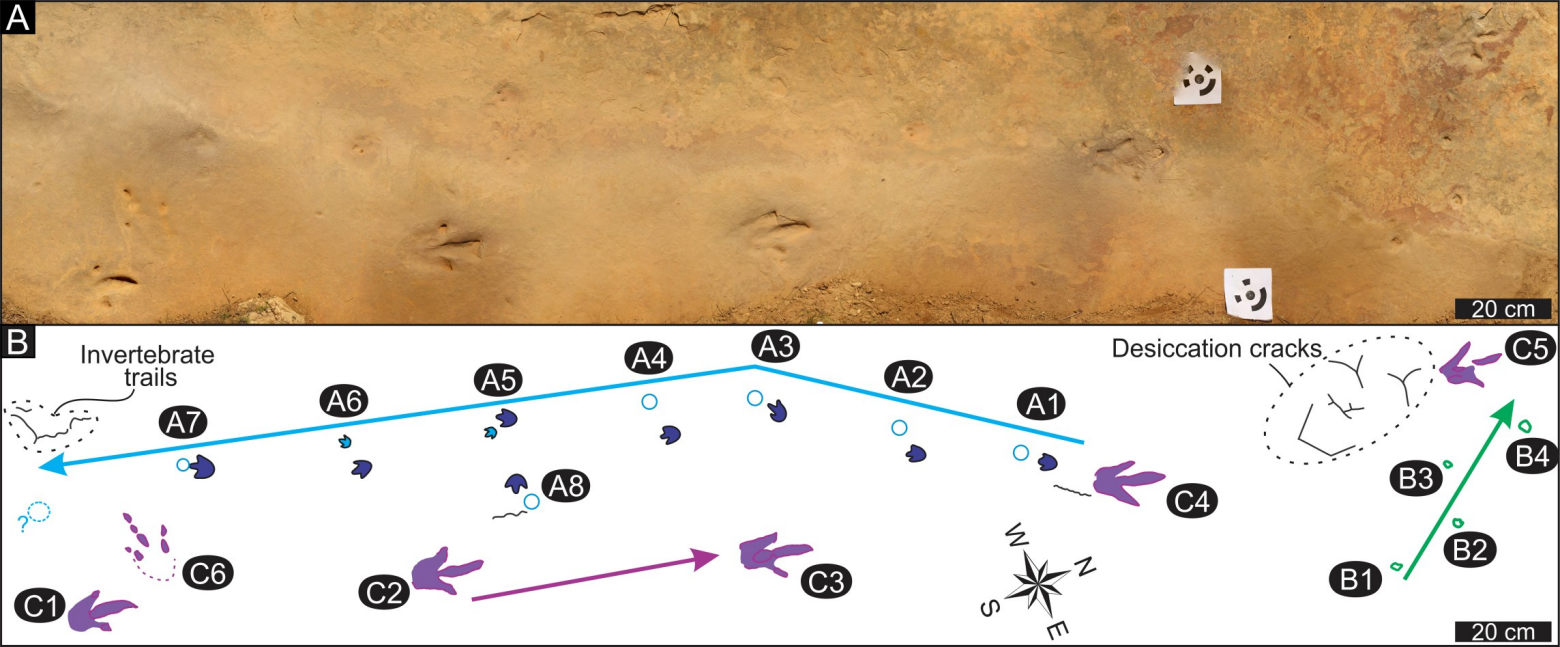
Trackways at the Highlands ichnosite. (A) Orthophotograph of the palaeosurface. (B) Interpretive outline map of the palaeosurface indicating the trackway patterns, invertebrate trails and desiccation cracks. Trackway A–blue; Trackway B–green; Trackway C–purple. Arrows show locomotion directions.

The Highlands ichnosite is the upper bedding plane of a <10 cm sandstone layer (see previous section) that contains a total of twenty-five tracks organized into five trackways (A-E; Fig 6). All tracks occur as negative epichnia along with invertebrate trails and desiccation cracks. Three different track morphotypes have been documented, which are described below as tracks A, B and C. Trackway E and D contain very poorly preserved tracks that are similar in outline to tracks in trackway B; they are not discussed further.

#### Tracks A

**Fig 7:**
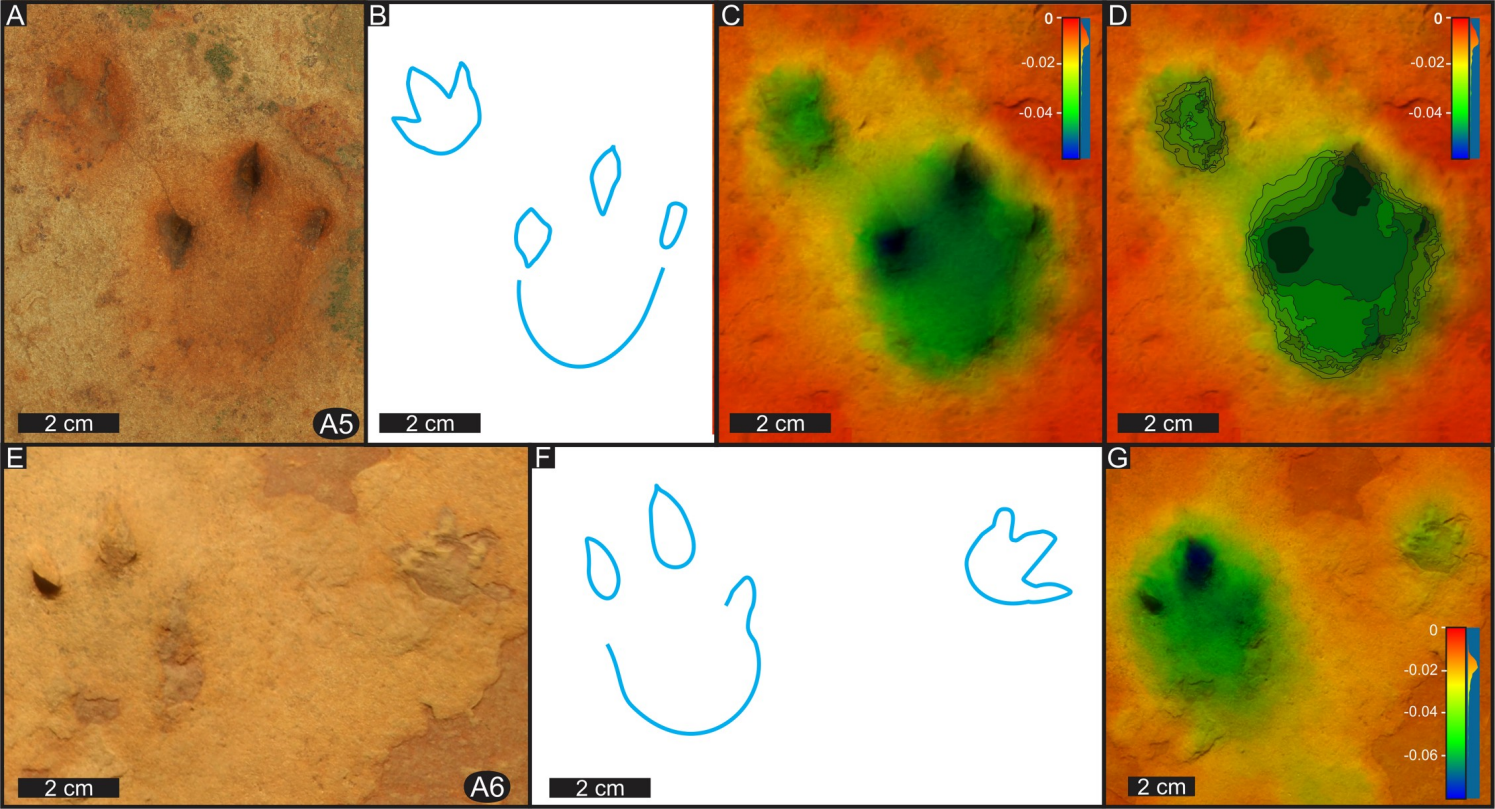
Details of individual manus-pes sets tracks in trackway A at Highlands. (A) Photograph of track A5. (B) Interpretive outline of track A5. (C) False-colour depth model of track A5. (D) Contour map of false-colour depth model of track A5. Contour-line intervals not regular. (E) Photograph of track A6. (F) Interpretive outline of track A6. (G) False-colour depth model of track A6. For relative position of tracks A5 and A6 within trackway A, see Fig 6. Depth scale in centimetres. Tridactyl outlines of manus tracks A5 and A6 are clearly seen in outcrop, although not apparent in 3D images, a difference due to lack of relief in the manus tracks.

**Fig 8:**
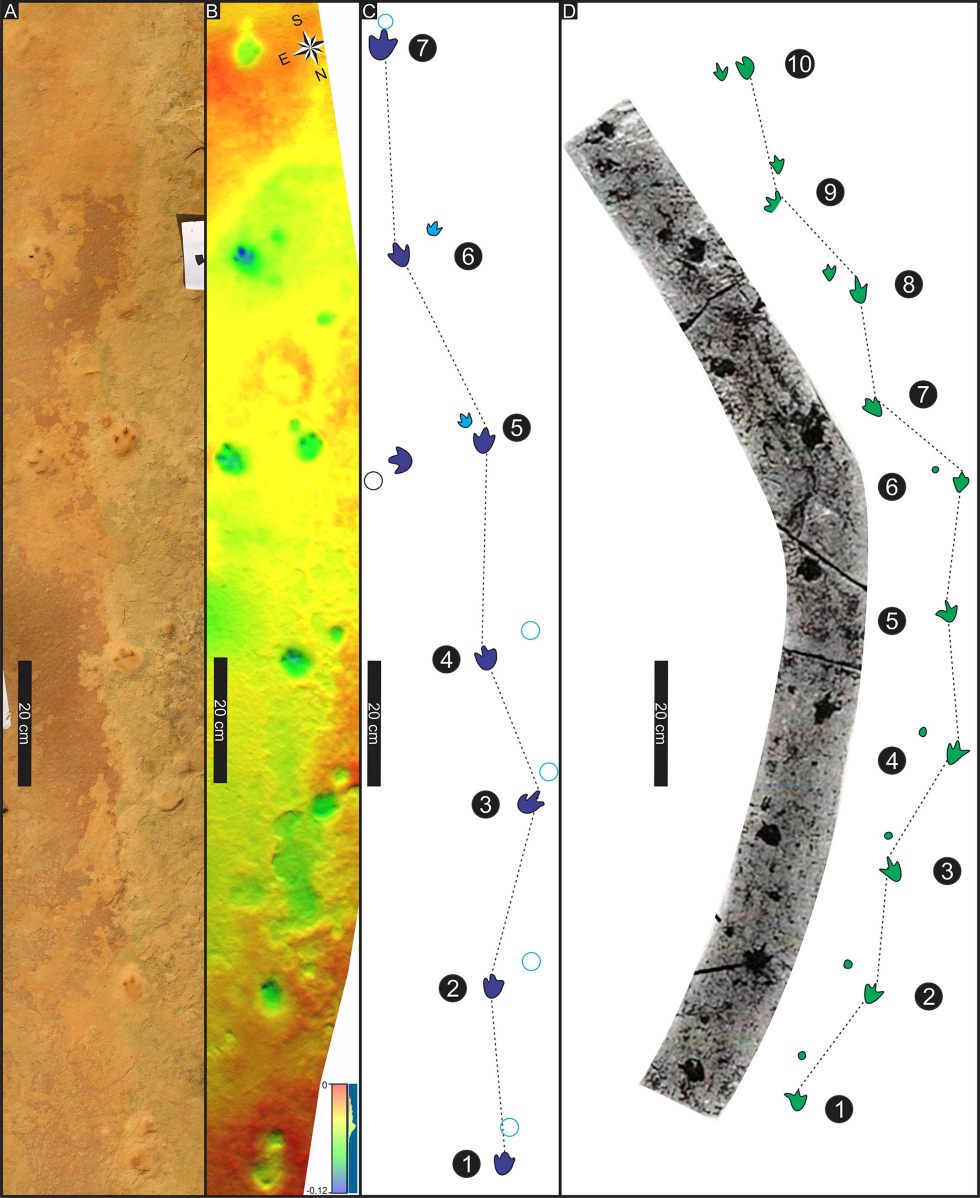
Trackway patterns of tridactyl quadrupeds from southern Africa. (A) Photograph, (B) False-colour depth model. (C) Interpretive outline of Highlands trackway A (holotype). (D) Photograph and interpretive outline of the Chewore trackway (paratype). Depth scale in centimetres. Locomotion direction toward top of the page in both trackways. Chewore trackway modified and republished from Lingham-Soliar and Broderick (2000) under a CC BY license, with permission from Taylor & Francis, original copyright 2000.

The new tetrapod ichnospecies *Afrodelatorrichnus ellenbergeri* erected here, and accommodated in the new ichnofamily Delatorrichnopodidae, is based on two highly distinctive trackways of small tridactyl quadrupeds: one, the holotype, from the Lower Jurassic (Pliensbachian-Toarcian) of South Africa (Figs 3, 7 and 8), and the other, a paratype, from the presumed Lower to Middle Jurassic of Zimbabwe (Figs 3 and 8). As the name suggests the distinctive *Afrodelatorrichnus* morphology is reminiscent of the ichnogenus *Delatorrichnus* from the Middle Jurassic of South America (Patagonia). Thus, there have been no previous reports of trackways of small quadrupeds, either obligate or facultative, in which both manus and pes tracks indicate tridactyly and in which the manus tracks registered on, ‘inside’ or medial to, rather than ‘outside’ or lateral to the trackway midline, as in the *A*. *ellenbergeri* configuration.

As discussed below, when the Zimbabwean trackway was reported, its ichnotaxonomic status was unclear due to several highly unusual features including the “strong negative (outward) rotation of the pes” and the “curious positioning of the manus prints in relation to the pes” ([74], p. 135). The recently-discovered South African trackway is remarkably similar to the Zimbabwean trackway, thus demonstrating that the Zimbabwean trackway is not an isolated example of anomalous trackmaker behavior or anomalous track registration conditions. On the contrary the two trackways mutually reinforce the evidence for a hitherto unnamed African ichnotaxon with multiple, and easily-diagnosed, morphometric features. We here recognize similar features in the South American ichnospecies *D*. *goyeneche* [77] from which we derived the ichnofamily name.

**Fig 9:**
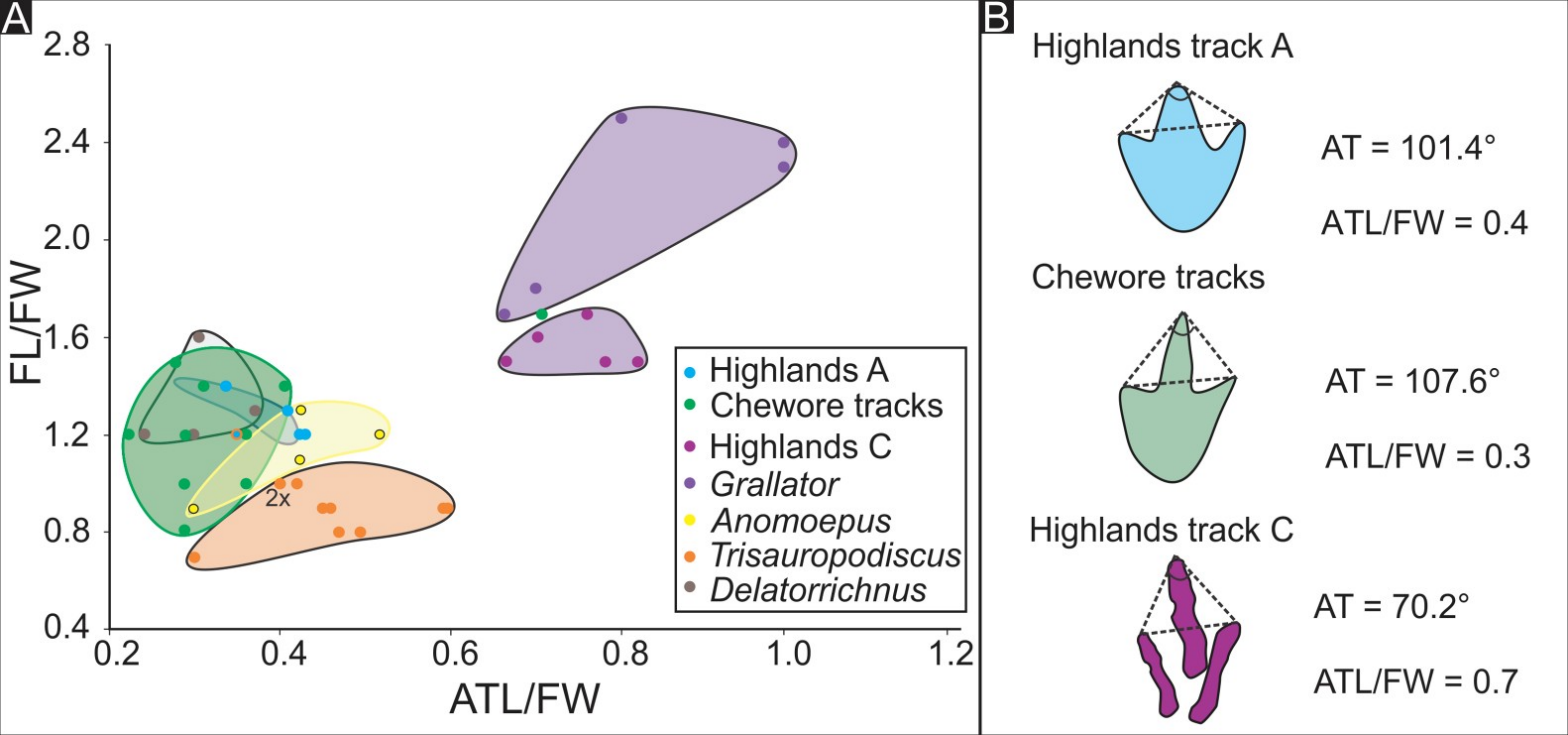
Analysis of the mesaxony of the Highlands tracks A and C as well as other selected Jurassic tridactyl tracks. (A) Bivariate plot of the footprint length to footprint width ratio (FL/FW) against the anterior angle length to footprint width ratio (ATL/FW). (B) Explanatory sketch of the measurements of AT used in (A). Data are from this study, Ellenberger [54], Lingham-Soliar and Broderick [74], Lockley [79], Dalman and Weems [80] and Abrahams et al. [81]. For raw data on the Highlands and Chewore ichnosites, see S1 Table.

In addition to confirming the marked similarities, highlighted by trackway morphometrics (see below, Figs 8 and 9, Table 1 and S1 Table), it is also important to note possible extramorphological preservation factors affecting the African and South American trackways. Some within or intra-trackway variation in preservation, number of preserved manus digit traces, and relative registration position of manus and pes may be extramorphological, or related to trackmaker changes in direction. For example, the *A*. *ellenbergeri* holotype trackway from South Africa preserves seven consecutive manus-pes sets, and shows only slight curvature to the left, whereas the paratype trackway from Zimbabwe reveals ten consecutive pes tracks with only eight corresponding manus tracks, and a more pronounced curvature to the left (Figs 3 and 7). These trackways can be compared with the *D*. *goyeneche* material which was re-described by de Valais [78] on the basis of the holotype trackway consisting of three manus-pes sets and additional paratype material.

**Table 1:** Ichnological measurements of Delatorrichnopodidae trackways.

Trackway parameter	*D*. *goyeneche*	*A*. *ellenbergeri* paratype Zimbabwe	*A*. *ellenbergeri* holotype South Africa
Pes footprint length FL	2.91	3.8	4.3
Pes footprint width FW	2.52	3.1	3.4
Pes FL/FW	1.15	1.3	1.3
Pes rotation	14°	17.8°	15°
Pes digit divarication	42°	50.4°	49°
Manus length	1.84	2.0	1.7
Manus width	1.29	1.6	1.9
Manus ML/MW	1.43	1.2	0.89
Manus rotation	-	-	21°
Pes pace (step)	-	20.6	30.0
Pes stride	29.4	38.9	57.8
Manus pace (step)	-	18.3	29.3
Manus stride	29.8	38.0	58.6
Pes trackway width	-	9.0	9.0
Pes pace angulation	162°	143°	154°
Manus trackway width	-	2.6	3.4
Manus pace angulation	171°	181.3°	181.2°
Estimated hip height T	13.1	17.1	19.4
Estimated hip height O	14.0	18.2	20.4
Estimated body L T	~ 34.0	~ 45.0	~ 51.0
Estimated body L O	~ 37.0	~ 48.0	~ 54.0
Estimated GA	~ 12.5	~ 25.0	~ 37.0

Measurements are in centimetres and degrees. Table is arranged from left to right in order of discovery and increasing size: from smallest, *Delatorrichnus goyeneche* [77, 78], to Zimbabwean *Afrodelatorrichnus ellenbergeri* [74] and largest, South African *A*. *ellenbergeri* (this paper). For raw data on the latter two, see S1 Table.

As noted above, it is well known that imperfect or extramorphological preservation may result in manus or pes tracks with less digit traces than the corresponding number of digits in the trackmaker. This problem that may apply to almost any trackmaker and its tracks in given circumstances. For example, *Anomoepus* and even some *Grallator* pes tracks are sometimes tetradactyl, indicating the registration of the hallux by functionally tridactyl trackmakers that registered tridactyl tracks in the majority of cases. A similar situation may apply to the registration of *Anomoepus* manus tracks, which, in the case of quadrupedal progression are ideally pentadactyl, but may be tetradactyl or even tridactyl. However, we know of no examples of *Anomoepus* trackways with between 7 and 10 consecutive manus-pes sets in which the manus prints with digit traces are consistently tridactyl, and occur with the other unusual and distinctive features of the *Afrodelatorrichnus* trackway such as strong heteropody and unusual placement of the manus (e.g., [18]). Moreover, it is contrary to the tenets of acceptable ichnological practice to infer the presence of digits that are not registered as traces. In this regard, even if one were to infer that the three manus digit traces (I-III) of *Afrodelatorrichnus ellenbergeri* described below represent a manus in which the shorter digits IV and V were not registered, in a predicated postero-lateral position it would still be possible, even reasonable, to infer that their failure to register was due to this same digit shortness (or digitigrade posture of digits I-III) which in itself is a morphological attribute that can affect track and trackway morphology. In short we must deal only with what is preserved, and part of a repeat pattern, and not what is not or might have been preserved. The only obvious exceptions are those of extreme extramorphological distortion of track morphology or cases where large samples allow convincing comparison between optimal and suboptimal preservation of well-known ichnotaxa.

### Delatorrichnopodidae ichnofamily nov.

**Diagnosis:** Trackway of a small obligate quadruped with tridactyl outwardly rotated pes and smaller manus with indistinct digit traces (Table 1 and S1 Table). Pes about twice as long and wide as manus, indicating moderate heteropody. Pes trackway width, greater than manus trackway width due to manus registration medial to trackway midline: i.e., typically inside pes tracks.

### Referred material

*D*. *goyeneche* [77, 78]

Unnamed trackway replica in the Bulawayo National Museum of Natural History of Zimbabwe (NMNHZ) and replica (GPIT1653) in the Geology Museum, University of Tubingen, Germany [74], here are designated as the paratype of *Afrodelatorrichnus ellenbergeri*.

The holotype of *Afrodelatorrichnus ellenbergeri*, trackway A from the Highlands locality in South Africa.

### *Afrodelatorrichnus* ichnogen nov.

**Referred material**: see paratype

**Holotype**: Trackway A from the Highlands locality in South Africa. Silicon rubber cast (BP/6/744) housed in the Evolutionary Science Institute at the University of the Witwatersrand (Johannesburg, South Africa).

**Type locality**: lowermost Lesotho Formation, Drakensberg Group (uppermost Pliensbachian-lowermost Toarcian, Lower Jurassic), ~ 45 m above the top of the Clarens Formation. Trackway site: GPS 28°24'53.50"S 28°15'11.14"E

**Paratype**: Unnamed Zimbabwean trackway cast from the ?Lower to Middle Jurassic post-Karoo Dande Sandstone Formation (Ntumbe River, Chewore South Safari Area, Lower Zambezi Valley, northern Zimbabwe) in the Bulawayo National Museum of Natural History of Zimbabwe (NMNHZ) and replica GPIT1653 in the Geology Museum, University of Tubingen, Germany. The radiometric age of the Ntumbe strata is unknown, and Early Jurassic to Early Cretaceous as well as Middle to Late Jurassic ages were suggested by Lingham‐Soliar and Broderick [74] and Ait‐Kaci Ahmed and Mukandi [82], respectively.

**Derivation of name**: referring to African trackways resembling *Delatorrichnus* from South America.

**Ichnogenus diagnosis:** Trackway of a small quadruped with tridactyl pes and ?tridactyl manus recording a distinctive gait in which outwardly rotated pes registered outside, or ‘lateral’ to, trackway midline and manus registered on or inside, or ‘medial’ to, trackway midline. Moderate heteropody, with pes more than twice and long and wide as manus. Marked contrast between moderately wide pes trackway width, more than twice pes width, giving pes pace angulation of about 150° compared with narrow manus trackway width and very high manus pace angulation > 180°.

### *Afrodelatorrichnus ellenbergeri* ichnosp. nov.

**Referred material**: as for ichnogenus, see paratype.

**Differential ichnospecies diagnosis:** Delatorrichnopodid trackways with outwardly rotated tridactyl pes, and ?tridactyl manus. Larger than, and differing from, *Delatorrichnus* in pes trackway width and lack of pes on manus overstep. Also differing from *Delatorrichnus* in configuration of pes pad impressions which are narrower and more widely separated in *Afrodelatorrichnus*.

**Holotype and paratype**: as for ichnogenus

**Type locality**: as for ichnogenus

**Derivation of ichnospecies name**: in honor of Paul Ellenberger, and his family’s extensive work on the history and prehistory of southern Africa.

**Description:** *Pes tracks* small tridactyl, mesaxonic and slightly elongate: mean length (L) 3.8–4.3 cm and mean width (W) 3.1–3.4 cm for the paratype and holotype trackways (N = 10 and N = 7) respectively; L/W = 1.3 in both cases. Pes mesaxonic with digit III longest and anterior triangle length to width ratio (ATL/AFW) 0.4–0.8, respectively. Pes rotation variable 0° to strongly negative or outward (*sensu* [83]: i.e., 0–34° and 0–45°, mean 15° and 17.8°, respectively. Digit traces deep, fusiform, lacking differentiated digital pad traces and sometimes separate from shallow, broad, posteriorly convex heel trace in holotype trackway. Mean divarication of digits II–IV is between 38° and ~ 50°, respectively.

*Manus tracks* small, tridactyl, mesaxonic, with occasionally clear digit traces: mean length (L) 2.0–1.7 cm and mean width (W) 1.6–1.9 cm for the paratype and holotype trackways (N = 8 and N = 7, L/W = 1.2–0.9), respectively. Thus, manus is about, or less than, half as long and wide as the pes, indicating clear heteropody. In the holotype trackway manus is situated anterior the pes in two cases, but well ‘inside’ (or medial to) corresponding pes in five other cases. The four most distal manus tracks are rotated positively (inward) at an average of 21° (N = 4). Likewise, the manus is situated anterior to, but well inside the pes in the paratype trackway. Rotation of manus only discernable in last three of paratype trackway manus-pes sets with sequential values of 22° (out), 15° (in) and 8° (out).

The holotype trackway consists of seven manus-pes sets, with and average left pes-right pes pace (PP) of 30.0 cm and corresponding pes stride (PS) of 57.8 cm. The mean step (PP) and stride (PS) values for the paratype trackway, made by a smaller animal (based on pes length) are 20.6 and 38.9 cm, respectively. The longer step recorded in the holotype trackways is reflected by a higher pes pace angulation (154°) compared with the paratype trackway (143°). However, the mean trackway width, 9.0 cm is the same in both trackways, and thus the mean pes trackway width is more than twice pes width in both trackways. The mean manus pace (MP) and stride (MS) in the holotype trackway are 29.3 cm and 58.6 cm, respectively with corresponding values of 18.3 cm and 38.0 cm for the paratype trackway. Thus, lengths of manus and pes steps and strides are similar. However, the mean manus trackway width is 2.6 and 3.4 cm, respectively, thus only slightly greater than actual manus track width. Such narrow manus trackway width values reflect the registration of the manus on or closer to the trackway midline than the pes, resulting in very high pace angulations up to 191° (means 181.2° and 181.3° for holotype and paratype, respectively). As noted below, values in excess of 180° indicate that the trackmaker registered footprints ‘inside,’ ‘across’ or medial to, the trackway midline.

#### Systematic discussion

As reviewed above, Lingham-Soliar and Broderick ([74], p. 135) noted the “strong negative (outward) rotation of the pes” and the “curious positioning of the manus prints in relation to the pes” described here in detail for the Zimbabwean trackway here designated as the paratype of *A*. *ellenbergeri*. More precisely, we can state that pes trackway width is more than twice pes width and that pes outward rotation, although variable, is as high as 45° and averages 15°–17.8° (Table 1). Likewise, the positioning of the manus is more accurately described as having registered on, medial to, or across the midline (i.e., crossing from lateral to or ‘outside’ the mid line to ‘inside’) with a tendency to inward rotation on the order of 21° in the holotype trackway. This inward pull on the manus creates very high mean pace angulation values (> 181°). In short, we can envisage small quadrupeds with a slight hind limb straddle and hind feet quite strongly rotated or ‘splayed’ outward. This hind limb “flat footed” straddle is accompanied by front limbs with little or no straddle and a strong tendency to register inwardly rotated manus prints medial to the trackway mid line. It is as if the gaits of the front and hind limbs were decoupled with polar opposite gaits. Thus, while the pes tracks registered consecutively on the left and right sides of the trackways, the manus tracks generally registered on the opposite sides. So left manus tracks are on the right side of the left pes tracks (e.g., in tracks 2, 4 and 6 of trackway A), and vice versa. However, while inside the pes tracks in most cases, they are generally within the outer pes trackway width (Fig 3).

There are noticeable and similar asymmetries in both trackways. In the holotype, South African *A*. *ellenbergeri*, the right side manus tracks (1, 3, 5 and 7) registered in front of, and close to the pes tracks (i.e., close to the line defining the right hand side of the outer pes trackway width), whereas the left manus tracks (2, 4 and 6) registered well to the right and almost twice as far away (Table 1): i.e., also close to the line defining the right hand side of the pes trackway width. This suggests the holotype trackmaker reached to the right with each left manus step, but actually veered slightly to the left while registering less right manus deviation. The picture is similar in the Zimbabwean trackway, but on the other side, with right manus footprints 2, 4, 6, 8 and 10 showing that the trackmaker reached and registered tracks well to the left of the corresponding pes footprints. This contrasts with the registration of the left manus footprints 1, 3, and 9 (5 and 7 are missing) with less, or no, deviation across the trackway midline. Still the trackway veers to the left as in the South African trackway, so there appears no obvious correlation between the curvature (veering) of the trackway and the difference in degree to which left and right manus tracks registered across (i.e., on the opposite side of) the trackway from the corresponding pes. Thus, while it is possible that the asymmetry of the trackways, reflecting and unusual gait is related to changes in direction, it is unclear why the Zimbabwean trackmaker turned about 40° to the left as the right manus consistently registered (veered) in that direction, and on the other hand the South African trackmaker veered almost 15° to the left, while its left manus reached and registered to the right side, away from the left veering direction. Thus, preferential veering of the front limbs to a given side did not correlate positively with changes in trackway to that same side.

We considered the possibility that the left and right manus tracks might have been reversed in our interpretation, thus implying a reversing of the manus rotation values. If this were the case, the manus tracks in the type trackway would all be in a straight line to the right-hand side of the line of the outer pes trackway width, while the track maker turned left and failed to register any tracks on the left side of the trackway. In the paratype trackway, the reverse would be the case with the poorly preserved manus tracks all closely aligned to with the left side of the line of the outer pes width for the first four manus-pes sets and then close to the trackway midline for the last three manus-pes sets. Regardless of which interpretation of manus sequence we chose (L-R-L-R-L etc. or R-L-R-L-R etc.), the manus tracks registered in a narrow line, on the right side in the holotype trackway, on the left side in the proximal part of the paratype trackway and in the middle of the distal part of the paratype trackway. The R-L-R-L-R etc. interpretation we suggest for the paratype trackway (Fig 3) places the manus trace just anterior to the pes, but the alternate interpretation of the manus (L-R-L-R-L etc.) decouples our interpretation of the “close” right manus-right pes configurations and places the left manus in front of the left pes track and vice versa in a distant left manus-right pes configuration. On could attempt to model the two aforementioned manus interpretations but without knowing the relative length of the trackmaker’s limbs or its identity, we consider this beyond the scope of this paper. All of these distinctive morphometric features, and the derived quantitative data (Table 1, Figs 3, 8 and 9) are important in demonstrating the uniqueness of *A*. *ellenbergeri* morphology with respect to tridactyl, pes and manus foot morphology, contrasting hind and front limb straddle, contrasting manus and pes rotation, and apparent potential for independence of left and right side manus registration patterns. Justification for these trackway configuration patterns as unique and ichnotaxonomically significant is supported by a comparative analysis between *A*. *ellenbergeri*, *D*. *goyeneche* and other potentially comparable ichnotaxa (Table 1, Fig 9).

#### Comparative analysis

Lingham-Soliar and Broderick ([74], p. 135) did not name the Zimbabwean trackway, here designated as the paratype of *A*. *ellenbergeri*. They did however make tentative comparisons with a number of ichnotaxa representing small and medium sized Late Triassic and Jurassic quadrupeds with tridactyl pes tracks. The best known of these is Late Triassic ichnogenus *Atreipus* represented by several ichnospecies [84], all of which have a *Grallator*-like pes with well-defined and well-differentiated digital pad traces. Pes tracks are typically 10–15 cm long with a tridactyl or tetradactyl manus typically situated anterior to or slightly antero-lateral to the trace of pes digit III. The manus is generally between one quarter and one third of the length of the pes, hence showing much greater heteropody than in the African trackways. Although there are no recorded trackways with more than three consecutive manus-pes sets [85], fig 3.12), *Atreipus* pes and manus tracks are consistently aligned in very narrow trackway with little or no pes rotation. Thus, there are almost no morphologically diagnostic points of comparison with the African trackways beyond the very general observation that the trackmakers were quadrupeds with a tridactyl pes.

*Anomoepus* is a relatively well known small to medium sized Early Jurassic ichnogenus representing a quadrupedal trackmaker. It belongs to the same ichnofamily (Anomoepidae, [86]) as *Moyenisauropus*, which was named from the Lower Jurassic of Lesotho by Ellenberger [54, 56]. Although known from many Lower Jurassic localities in New England where *Anomoepus* was first described [87] as well as localities in other regions such as the western United States [88], there are almost no trackways known that indicate sustained quadrupedal progression where more than two or three consecutive manus-pes sets were registered. All diagnostic pes tracks are tridactyl or tetradactyl with small antero-medially oriented digit I (hallux) trace, and the manus tracks are pentadactyl and outwardly rotated. The longest recorded trackways indicate bipedal progression with slight inward rotation of the pes [18, 88]. This suggests that the *Anomoepus* trackmaker was a facultative biped that progressed bipedally more often than quadrupedally. Most manus tracks appear to be associated with sitting or squatting behavior. Thus there are multiple diagnostic features that differentiate *Anomoepus* from the African trackways described here.

Lingham-Soliar and Broderick ([74], p. 135) referred to the enigmatic ichnospecies *Delatorrichnus goyenechei* named by Casamiquela [77] from the Middle Jurassic but said little to suggest an ichnotaxonomic affinity with the Zimbabwean trackway. Since 2000 *D*. *goyenechei* has been re-described by de Valais ([78], p. 30) and shown to represent a small obligate quadruped “with symmetric manus and pes impressions, with manus impressions located medial to, and usually in contact with the pes prints.” In some case, the pes overprints the manus. This description together with the statement that the pes prints are tridactyl and display “outward rotation relative to the midline (mean 14°)” (op. cit. p. 31) suggest a number of significant similarities with the African trackways. However, de Valais [78] did not compare *D*. *goyeneche* with the Zimbabwean trackway, and the South African trackway was not then known.

The respective mean pes length and width (2.91 cm and 2.52 cm = L/W 1.15) of *D*. *goyeneche* indicate a relatively small trackmaker with a pes about 77% as long and 91% as wide as the Zimbabwean trackway, and about 68% and long and 74% as wide as South African trackway. However, there are also differences, the most evident being the narrower trackway in *D*. *goyenechei* with correspondingly higher pes pace angulation (162°) and lower manus pace angulation (171°), the latter due to the lesser tendency of the manus to register across the midline, away from the pes. This in turn appears to result in a consistent pes on manus overstep, not seen in the African trackways. Despite suggestions by de Valais ([78], p. 31) that “pedal digit impressions are relatively thick and lack digital pad traces” some *Delatorrichnus* specimens including MLP 65-XI-12-1/1, and the holotype (MLP 60-X-31-6) (op. cit., fig 2.1 and 2.2) clearly show at least two separate, and certainly “thick” or fleshy digital pads on each of digits II, III and IV. Another distinctive feature is that the distal pad of pes digit III appears wider than the remainder of the digit traces. Although this may be a preservational phenomenon related to lack of confinement of digit III, by adjacent digits II and IV, during registration [89], the distal traces of *Afrodelatorrichnus* are far narrower than in *Delatorrichnus*.

The mean stride length of *D*. *goyenechei* is 29.42 cm compared with 57.8 and 38.9 cm for the South African and Zimbabwean trackways, respectively. The *D*. *goyenechei* manus is poorly known due to sub-optimal preservation but it is stated (op cit. p. 31) that there are four digits (I–IV) with III > IV > II > I, and a mean overall length and width of 1.84 and 1.29 cm, respectively. Thus, ostensibly the manus is 92% as long and 81% as wide as the Zimbabwe manus and 108% and 68% as long and wide as the South African trackway. It is important to note that the size, shape and number of manus digits represented in *D*. *goyeneche* and the two *A*. *ellenbergeri* trackways is not entirely clear in most cases due to suboptimal preservation of such small tracks. In the case of the former ichnospecies, overprinting also obscures manus track morphology in most cases.

De Valais [78] commented (op cit. p. 32) that “At present this ichnospecies has not been ambiguously recorded at any other locality.” She did not refer to the Zimbabwean trackway reported by Lingham-Soliar and Broderick [74]. Instead she cited (op. cit. p. 30) “a manus-pes set from the Early Jurassic Zagaje Formation of Sołtykow, Poland, … compared with or included in *Delatorrichnus* [by] [90–92]” and went on to suggest that the trackmaker was most likely a small ornithischian, an interpretation also supported by Abrahams et al. ([81], p. 971) who suggested a “heterodontosaurid trackmaker.” The tracks from Poland labelled as *Delatorrichnus* isp. ([91], p. 208) have “a tridactyl manus (1.6 cm long and 2 cm wide) with tridactyl pes (5 cm long and 4 cm wide)” were described as “*Atreipus*-like” and “similar” to the Zimbabwean tracks. See Gierliński and Niedźwiedzki [90] for further suggestions of *Atreipus* occurrences in the Lower Jurassic, in contrast to the typical Late Triassic occurrences [84].

Estimation of the trackmaker gleno-acetabular (GA) distance using the method of Leonardi ([83], Pl. VIII C) gives an estimated GA or trunk length of 25 cm for the Zimbabwean trackmaker and 37 cm for the trackway A trackmaker (GA estimates assume no period of ‘suspension’ when all feet lost contact with the substrate). Thus, in linear dimension the trunk of the latter animal was 50% longer. The GA estimate for *D*. *goyeneche* from South America, based on measurements for photographs of the type trackway ([78], fig 2) is 12.5 cm. Using the ratio (r) between footprint (foot) length and height at the hip (h) for small theropods (r = 4.5) or small ornithopods (r = 4.8) proposed by Thulborn [59], we can estimate the hip heights for the trackmakers of the two *A*. *ellenbergeri* trackways at between 17.1 and 18.2 cm for the theropod model and between 19.4 and 20.4 cm for the ornithopod model. Likewise, the respective values for the *D*. *goyeneche* trackway are 13.1 and 14 cm. Using the hip height to total body length ratio of 2.63 estimated for theropods [93], we can estimate body lengths of between ~ 45 and 48 cm or ~ 51 and 54 cm (for theropods or ornithopods, respectively) from the two African trackways, with corresponding body length estimates of ~ 34 –~ 37 cm from the *D*. *goyeneche* trackway.

As noted above there are no reported *Atreipus* or *Anomoepus* trackways that reveal more than three consecutive manus-pes sets. In the case of *Atreipus*, it is thus difficult to determine whether the trackmaker was an obligate or habitual quadruped. In the case of *Anomoepus*, it is generally inferred that the trackmaker was a facultative biped, that progressed bipedally more often than not. This indicates that *A*. *ellenbergeri* was most likely an obligate quadruped, certainly more obligate than the *Anomoepus* trackmaker. The two manus tracks (5 and 7) ‘missing’ from the Zimbabwean trackway might be explained one of three ways: as the result of poor preservation, around the time of trackway registration (or post-exhumation), as the result of a brief switch from quadrupedal to bipedal and back to quadrupedal progression, or as the result of pes overstep on manus [78, 94]. Although there is a slight increase in step and stride length between tracks 3 and 7, the increase is not confined to the 5–7 interval. Thus there is no obvious change in step and stride to support a marked change in gait.

Following Thulborn’s [59] formula, the allometric gait values range between 2.7 to 3.5 for Trackway A, indicating that the trackmaker’s locomotion transitioned from a “trotting” gait to a “running” gait. The allometric gait of the Zimbabwean trackway ranges between 2.1 to 2.8, indicating a predominantly “trotting” gait. The calculated allometric and morphometric speeds for trackway A are 2.6 and 2.3 m/s, respectively, and 1.6 and 1.4 m/s for the Zimbabwean trackway, respectively.

Regarding trackmaker identity, there are no universally recognized theropod trackway morphotypes (ichnotaxa) that indicate obligate quadrupeds. On the contrary, the only trackways that include manus tracks are associated with crouching or squatting behavior (see [95] for review). The only possible exception appears to be the *Atreipus* trackmaker with its *Grallator*-like pes. This ichnogenus was attributed to an ornithopod by Olsen and Baird [84], but to a theropod (coelurosaur) by Thulborn [59] who also discussed the early debate about *Delatorrichnus*, opining (op. cit., p. 161) that “*Atreipus* and *Delatorrichnus* seem to be remarkably similar aside from differences in preservation and the slightly greater size of manus prints in the latter.” (presumably meaning greater proportional size, i.e., lesser heteropody). Thulborn (op. cit., p. 161) went on to suggest that “some early coelurosaurs were in the habit of moving around on all fours.” However, although Thulborn’s inference that the *Delatorrichnus* trackmaker was a theropod is consistent with the original interpretation of Casamiquela [77], the reinterpretation of the type material by de Valais [78] suggests an ornithischian trackmaker, as noted above. This interpretation is in part supported by the observation that “pedal digital impressions are relatively thick” ([78], p. 31), presumably meaning unlike the sequence of digital pads comprising slender digit traces as in *Grallator* and *Atreipus*. However, as noted above at least two thick pads can be discerned in *Delatorrichnus* pes digit traces II, III and IV, although admittedly this 2-2-2 pad formula is not like the typical 2-3-4 theropod formula. We should stress that we do not consider *Atreipus* and *Delatorrichnus* “remarkably similar.” Beyond significant size and pes rotation differences, the theropod like pes, with the separate, 2-3-4 digital pad configuration in *Atreipus* is wholly unlike *Delatorrichnus*.

Trackways attributed to ornithischians may indicate obligate bipeds, facultative bipeds or obligate quadrupeds. Common features of most diagnostic ornithischian ichnotaxa are pentadactyl manus tracks, although they may show less than five digit traces if poorly preserved or reflective of digits bound together by integument. Most ornithischian pes tracks are tridactyl without discrete digital pad traces, as in most Cretaceous ornithopod pes tracks (which are inwardly rotated) and the purported stegosaur track *Deltapodus*. However, some pes tracks are tetradactyl as in the purported hypsilophodontid track *Hypsiloichnus* [96] and the ankylosaurian tracks *Tetrapodosaurus* [97].

The features common to *D*. *goyeneche* and *A*. *ellenbergeri* (Delatorrichnopododae) are a tridactyl pes with outward rotation and without discrete digital pad traces, a smaller manus with at least three digit traces and strong indications that the trackmakers were obligate quadrupeds. The manus is also often situated inside the pes, as occasionally seen in large ornithopods [98], which can also overprint manus on pes as in the case of *Delatorrichnus* (cf. [78]). Thus, on balance we infer that the Delatorrichnopododae represents small, ornithopod-like ornithischians, with fleshy, thickly-padded feet.

To date only three trackways assigned to Delatorrichnopodidae ichnospecies have been reported, two from the ?Lower to Middle Jurassic of southern Africa, and one from the Middle Jurassic of South America (Patagonia). This allows for speculation that the ichnofamily may have had its origins and main distribution in Gondwana. Reports of isolated tracks representing this ichnofamily (i.e., *Delatorrichnus* isp.) from Europe are based on limited, sub-optimally preserved material. Thus, more well-preserved delatorrichnopodid trackway material is needed to facilitate detailed comparison with the southern hemisphere material.

Given the relative abundance and global distribution of the theropod tracks *Grallator* and *Eubrontes* and the ornithischian track *Anomoepus* giving rise to the suggestion of a Lower Jurassic tetrapod tracks biochron [99], the recognition of delatorrichnopodid tracks points to the recognition of comparatively rare evidence for a distinctive small ornithischian.

#### Tracks B

**Fig 10:**
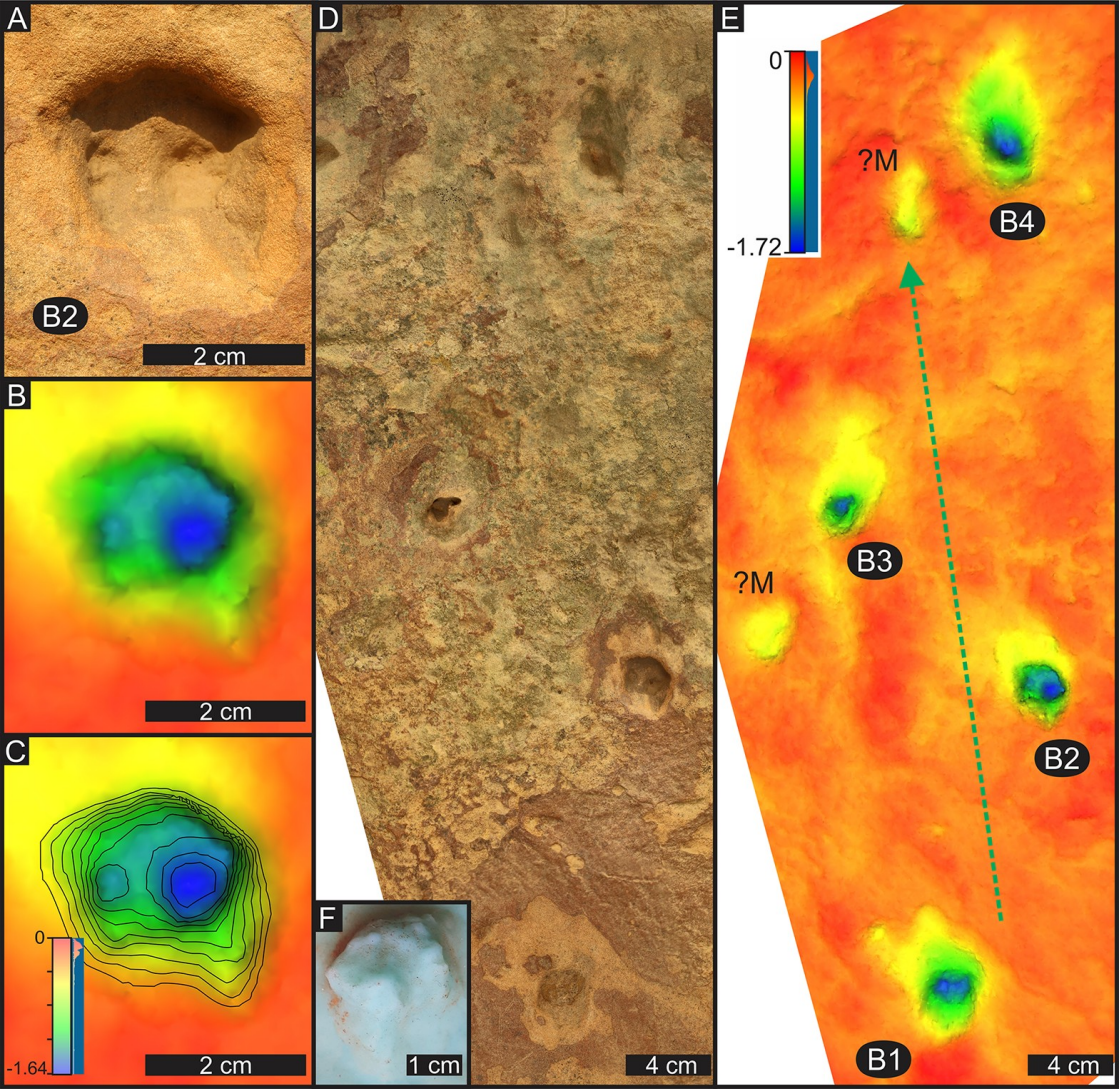
Details of track B2 and trackway B at Highlands. (A) Photograph of track B2. Note that shadow obscures the toe marks (compare to F). (B) False-colour depth model of track B2 (depth map). (C) Contour map of the false-colour model of track B2. (D) Photograph of trackway B. (E) False-colour depth model of trackway B. Possible manus tracks are labelled ?M. Depth scale shown in C is applicable to B as well. For relative position of track B2 within the trackway, see D and E. For relative position of trackway B within the palaeosurface, see Fig 6. Green arrow shows the locomotion direction. Depth scales in centimetres. (F) Photograph of the silicon rubber cast with inferred tiny circular digit marks and arc (see text explaining the tentative nature of these features).

These are eleven, poorly preserved, deep tracks of a small animal with slightly elongated feet with an average length (FL) of 2.8 cm and average width (FW) of 2.3 cm (FL/FW = 1.2 for the eight best preserved tracks; Fig 10; Table 2). It appears the tracks are deeper anteriorly, with extremely faint, short toe traces, inferred from close inspection of tracks and silicon rubber casts (Fig 10A–10C and 10F). The inferred toe traces appear obscured and modified by original preservation conditions and/or post-exhumation erosion. Possible manus tracks cannot be ruled out but that their positions are somewhat irregular, and only show up, albeit vaguely, on the depth map of trackway B as indistinct, slightly oval, faint impressions (Fig 10E).

**Table 2:** Ichnological measurements of trackways B, D and E at Highlands.

Trackway	Footprint No.	FL	FW	FL/FW ratio	PP		PS		P ANG	PR	Digits (Y/N)	No. of P digits	PTW
**B**	1	2,4	2,4	1,0	1 to 2	17	1 to 3	24	116	-1	unclear	unclear	9,3
	2	3	2,2	1,4	2 to 3	13	2 to 4	24	106	-1	unclear	unclear	10,5
	3	1	1,8	0,6*	3 to 4	16	–	–	–	-1	yes	3 or 4	11,6
	4	2,5	1,7	1,5	–	–	–	–	–	unclear	unclear	unclear	–
	**Average**	**2,2**	**2,0**	**1,0**	** **	**15,3**	** **	**24,0**	**111,0**	** **	** **	** **	**10,5**
**D**	1	*3	3,2	0,9	1 to 2	22	1 to 3	38,7	–	–	unclear	unclear	–
	2	3,4	2,3	1,5	2 to 3	20	–	–	–	–	unclear	unclear	–
	3	3,6	2,8	1,3	–	–	–	–	–	–	unclear	unclear	–
	**Average**	**3,5**	**2,8**	**1,2**	** **	**21,0**	** **	**38,7**	** **	** **	** **	** **	** **
**E**	1	2,4	2,2	1,1	1 to 2	27	1 to 3	51,1	–	–	unclear	unclear	–
	2	*1.8	2,3	0,8	2 to 3	30	2 to 4	43,2	–	–	unclear	unclear	–
	3	2,4	2,1	1,1	3 to 4	22	–	–	–	–	unclear	unclear	–
	4	2,6	2,4	1,1	–	–	–	–	–	–	unclear	unclear	–
	**Average**	**2,5**	**2,3**	**1,0**	** **	**26,3**	** **	** 47,2**	** **	** **	** **	** **	** **

Measurements are in centimetres. From left to right (Trackway): each trackway is labelled with a letter (e.g., B, D, E). (Footprint No.): number of pes mark in the trackway. (FL): pes length. (FW): pes width; (FL/FW) foot length to width ratio; (PP): pes pace; (PS): pes stride; (P ANG): Pace Angulation for pes; (PR): pes rotation, which is the orientation of the longitudinal axis of the pes respect to the trackway midline; rotation is expressed in qualitative terms: 0 (zero) = no rotation, 1 (one) = inward rotation, -1 (minus one) = outward rotation of pes; (Digits Y/N): presence (yes: Y) or absence (no: N) of digit marks on the footprint; (No. of P digits): number of visible digit marks on the foot mark; (PTW): pes trackway width.

*: incomplete track measurement.

Dash: measurement could not be taken.

The eleven tracks form three trackways (B, D, E; Table 2), consists of four, three and four tracks, respectively. Assuming B1, B2, B3 and B4 represent a L-R-L-R sequence in trackway B, there appears to be an alternating long-short-long step sequence (Fig 10E). The presumed manus and pes tracks are typically difficult to differentiate, possibly due to overprinting. The average trackway width is ~ 10.5 cm, which is > 4.5 times wider than the average pes width. Pes rotation is negative and the average pes angulation is 111°. Additional trackway parameters (Table 2 and S1 Table) are uncertain, and speed calculations are not given here due to reservations about trackway configuration. For these reasons, labelling tracks B as *Brasilichnium*-like is also very tentative, nonetheless consistent with the interpretation of a *Brasilichnium*-like track, we see an arc of inferred toe traces with the inter toe trace (~ digit I) in the predicted posterior position (Fig 10F; see below).

It is difficult to determine whether the trackmakers of tracks B, D and E were facultative quadrupeds or obligatory bipeds, because manus prints are difficult to interpret. However, the relatively wide trackways (PTW in trackway B: 10.5 cm) relative to track size argues in favor of a small quadruped with a wide straddle. There are no known examples of well-defined small trackways of obligatory bipeds with these dimensions. For example, the pace angulation value of 111° in trackway B indicates an intermediate limb posture between cursor (‘fully erect’) and noncursor (‘sprawlers’) tetrapods [100]. Taxa with similar pace angulations are sprawling lizards, and semi-erect, high‐walking crocodilians as well as ‘noncursorial’ (didelphids, murids and mustelids) mammalian groups [100].

**Fig 11:**
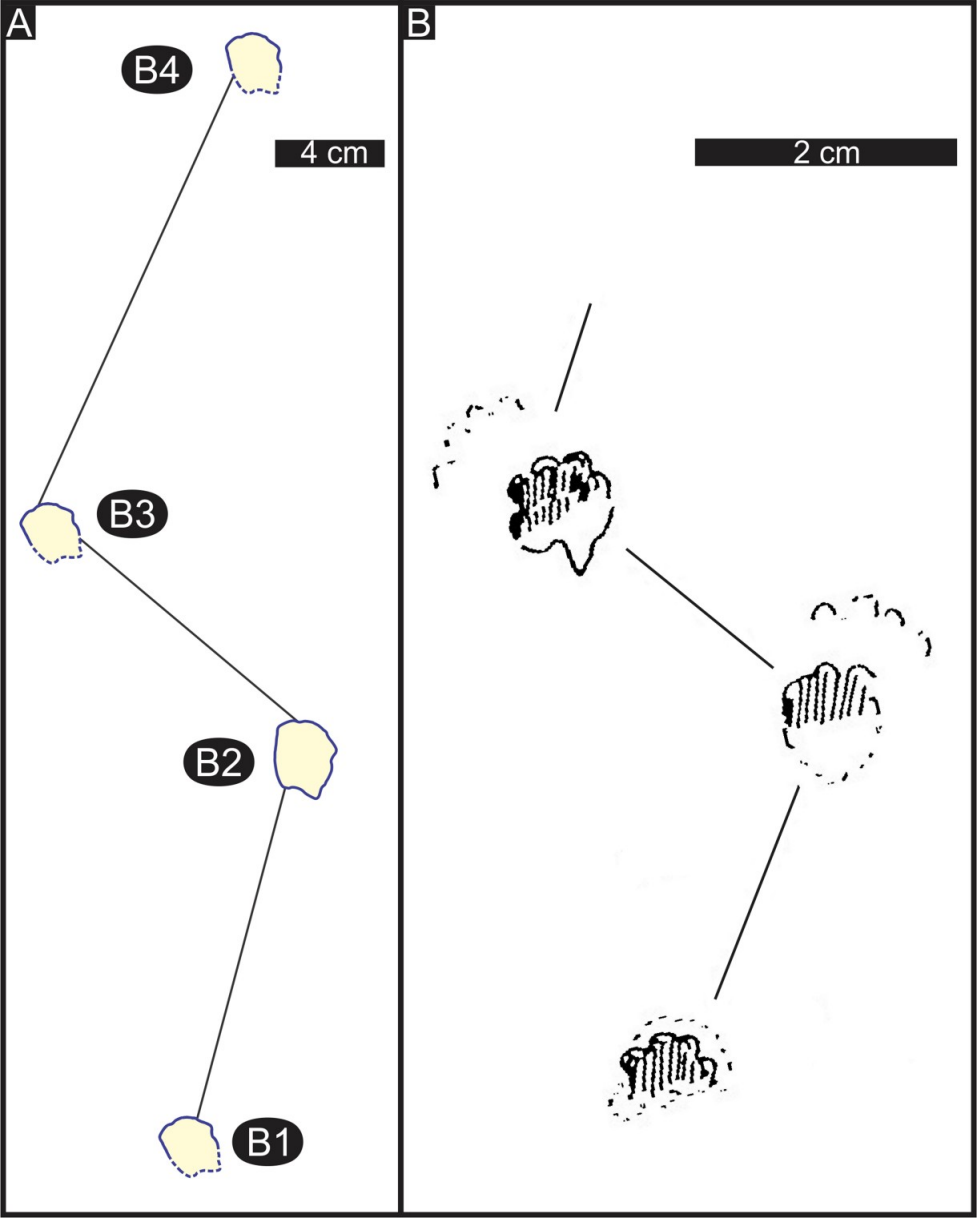
Comparison of Early Jurassic trackway patterns from southern Africa. (A) Schematic illustration of Highlands trackway B from the lower Lesotho Formation, Drakensberg Group (south of Bethlehem, South Africa). (B) *Eotetrapodiscus cursor* from the upper Elliot Formation, Stormberg Group (zone B1, lower Moyeni, Lesotho). Fig B modified and republished from Ellenberger ([54], fig 72) under a CC BY license, with permission from the Geological Society of South Africa, original copyright 1970.

Among the southern African Jurassic ichnites, the overall shape of the track B is slightly similar to *Eotetrapodiscus cursor* ([54, 56], fig 72), which was defined from an ichnite made by a small animal with cursorial progression in the upper Elliot Formation (zone B1; Lower Jurassic) at lower Moyeni in Lesotho (Fig 11). This trackway is figured as comprising three consecutive pes impressions with four digits each, pace angulation of 107° and with an overall trackway configuration that is somewhat similar to trackway B. However, *Eotetrapodiscus is* ~ 2.8 times smaller than the Highlands tracks B, being only ~ 1-cm long and ~ 0.8-cm wide. Although, Ellenberger [58] linked *Eotetrapodiscus* to basal mammals (e.g., *Erythrotherium*, *Megazostrodon)*, in a re–evaluation of some of the Lesotho ichnotaxa, Lockley et al. [57] classified *Eotetrapodiscus cursor* as possible mammal/protomammal tracks, but did not infer more specific affinities. D'Orazi Porchetti et al. ([101], p. 25; [101], p. 12) mentioned the similarity of *Eotetrapodiscus* to *Brasilichnium*, both of which have been interpreted as tracks of small mammaliaforms.

Made by small tetradactyl quadrupeds of mammaloid affinity with wide feet and short toes, *Brasilichnium*-like tracks have rounded to transversely oval pes shape, marked heteropody and occur in Late Triassic–Late Cretaceous aeolian dunes and non-aeolian deposits of South and North America as well as Namibia (e.g., [101–106]). A larger form of *Brasilichnium* has recently been named both as *B*. *anaiti* by D'Orazi Porchetti et al. [105] and as *Aracoaraichnium leonardii* by Buck et al. [107], however Francischini et al. [106] assert that these ichnotaxa are “junior subjective synonyms” of the Early Jurassic *Navahopus falcipollex* (e.g., [102, 108, 109]).

Tracks B, D and E at Highlands show some resemblance to *Brasilichnium*-like tracks that have been described, particularly, from the Toarcian of North America (e.g., [103]) and Lower Cretaceous of Namibia (e.g., [104]). This similarity includes the overall track shape, arc-like arrangement of the four digits, indistinct manus tracks, trackway width, pace angulation, etc., but most of all the preservation of a transverse ridge (‘sand crease’), which is the mould of the digital arcade between the digit marks and sole (metatarsals) print. This anatomical feature along the metatarsophalangeal line or metapodial-phalangeal line is typical in the feet of mammaloids [110]. Tracks B, D E appear more elongate than typical *Brasilichnium* because they are deeper.

Although, strong heteropody is a hallmark feature of *Brasilichnium*, manus tracks are paradoxically less commonly preserved relative to pes tracks. For example, in a sample of 669 pes prints of *Brasilichnium elusivum*, only 20% of the tracks were associated with manus prints, and only 10 out of 68 trackways with walking gaits showed manus traces ([101], p. 17). The abundance of pes-only tracks has been attributed to, among others, the lower preservation potential of the smaller and lightly impressed manus on primary surfaces (and thus lack in undertrack assemblages) and overprinting of manus tracks by the pes (e.g., [102, 111]). Furthermore, substrate related influences (e.g., inclination of the sediment surface) may also influence the preservation (e.g., [103, 104]). Indeed, the lack of definite manus tracks in the trackway B, and their apparent lack in trackways D and E may be a consequence of the mode of movement by small tetradactyl quadrupeds across the wet/damp sand in flat ephemeral stream bed at Highlands, which was different to that on sloping foresets in dry aeolian sand elsewhere. In the case of inclined and relatively unstable sandy slopes, the animals probably had to place greater weight on their manus to maintain balance, hence the manus preservation in the latter case is more common.

To sum up, based on the trackway B parameters at Highlands, other Drakensberg Group trackways (e.g., [54]) and the ichnological and osteological record of the conformably underlying Lower Jurassic Stormberg Group (e.g., [112]), the trackmakers of trackways B, D and E were possibly small mammaliaforms with a semi-erect leg posture. However, this attribution is tentative and a more explicit identity of the mammaliaform trackmakers (e.g., tritylodonts, trithelodonts, true mammals) awaits the discovery of better-preserved materials.

#### Tracks C

**Fig 12:**
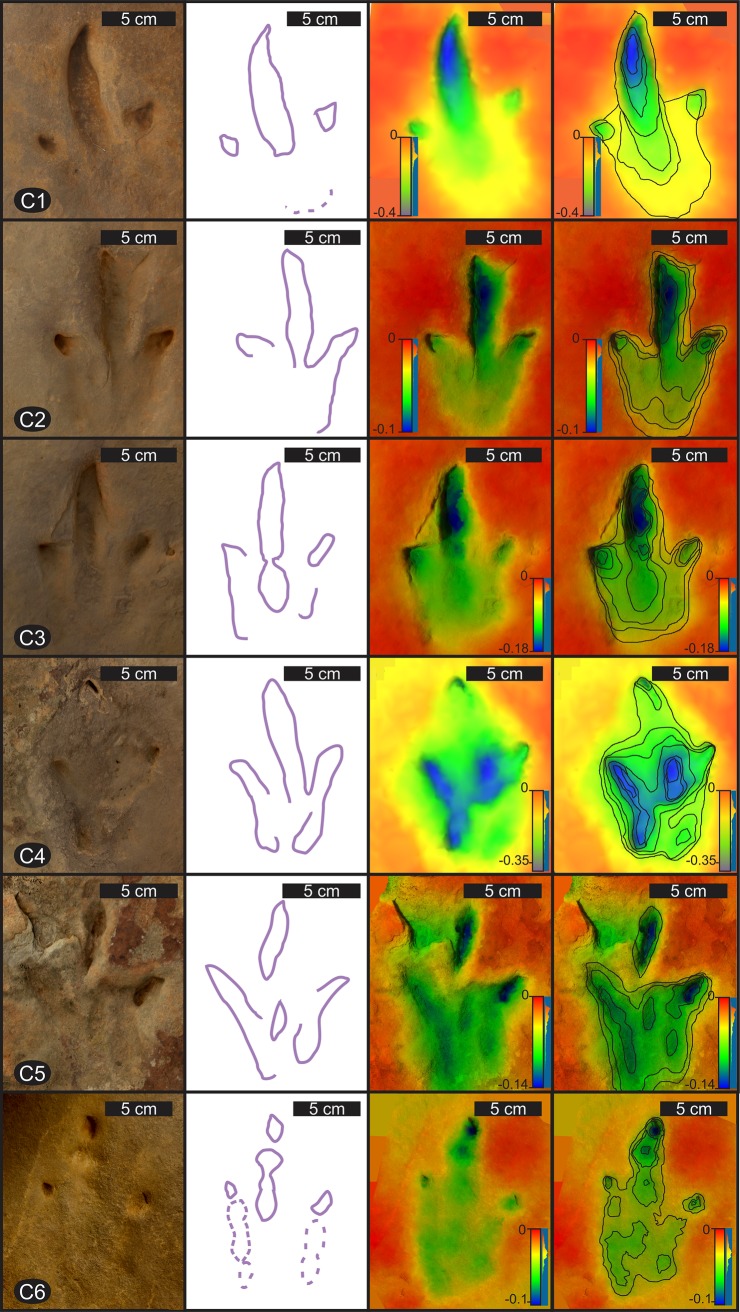
Details of tracks C1–6 at Highlands. Each row shows from left to right: photograph; interpretive outline from photograph; false-colour depth model and contour map superimposed on false-colour depth model. Depth scale in millimetres; for clarity, contour-line intervals not regular. Odd numbers (C1, C3, C5) are left footprints, even numbers (C2, C4) are right footprints. C6 belongs to a different trackway, and has clearer discrete digital pad traces compared to C1–5. For relative position of tracks within main the trackway, see Fig 6.

All tracks C are elongate tridactyl pes tracks with lengths (average FL = 13.6 cm) greater than widths (average FW = 8.7 cm) with a mean FL/FW ratio of 1.6 (Table 3; Figs 6 and 12). The tracks are mesaxonic, with digit III lengths being the longest (average 11.6 cm), having an average ATL/FW of 0.8 (Fig 9). Total digit divarication (II^IV) is variable, ranging from 29–48° with a mean of 38° (Table 3). These digitigrade tracks have gracile, slightly curving digits, which terminate in pointed tips with some tracks preserving claw mark impressions (e.g., C1, C2, C3; Fig 12). Excluding track C4, which is a poorly preserved track, the digit tips are very deeply impressed (Figs 6 and 12). Faint digital pad impressions can be observed in tracks C3 (digit III) and C6 (digits II, III and IV; Fig 12).

**Table 3:** Ichnological measurements of trackway C at Highlands.

Footprint No.	FL	FW	FL/FW ratio	PP		PS		P ANG	PR	Digits (Y/N)	No. of P digits	LII	LIII	LIV	TRp	OTW
1	*12,5	8,5	1,5	1 to 2	71	1 to 3	140	185	1	Y	3	–	*9,4	–	5	16,0
2	14,2	9,2	1,5	2 to 3	68	2 to 4	142	190	1	Y	3	8,6	12,8	10,9	6,5	15,5
3	14,2	8,5	1,7	3 to 4	75	3 to 5	148	173	1	Y	3	8,4	12,6	8,9	2,9	15,7
4	14,2	8,8	1,6	4 to 5	74			–	-1	Y	3	7,7	–	–	9,1	15,9
5	13	8,5	1,5					–	1	Y	3	6,4	–	7,1	7	–
*Average*	13,6	8,7	1,57		72,0		143,33	182,7				7,8	11,6	9,0	6,1	15,8

Measurements are in centimetres. From left to right: (Footprint No.): number of pes mark in the trackway. (FL): pes length. (FW): pes width; (FL/FW) foot length to width ratio; (PP): pes pace; (PS): pes stride; (P ANG): Pace Angulation for pes; (PR): pes rotation, which is the orientation of the longitudinal axis of the pes respect to the trackway midline; rotation is expressed in qualitative terms: 0 (zero) = no rotation, 1 (one) = inward rotation, -1 (minus one) = outward rotation of pes; (Digits Y/N): presence (yes: Y) or absence (no: N) of digit marks on the footprint; (No. of P digits): number of visible digit marks on the foot mark; (LII–IV): Length of respective pes digits; (TRp): Track rotation for pes (quantitative); (OTW): outer trackway width.

*: incomplete track measurement.

Dash: measurement could not be taken.

Five of C tracks form one narrow trackway that is 3.3 m long (Fig 6) with an average pes-pes pace (PP) of 72 cm and corresponding pes stride (PS) of 143 cm (Table 3). The average pace angulation is 182°. Four tracks (C1–3, C5) are rotated positively, but only slightly (inward, towards the trackway midline) at an average of ~ 5.5° (N = 4), with the distal traces of digit III also inwardly rotated in typical theropod configuration. The average outer trackway width is 15.8 cm, and thus the mean trackway width is ~ 1.8 times wider than the average pes width (Table 1). Overall the morphological quality of the tracks decreases along the trackway, and manus tracks are not discernable on the palaeosurface (Fig 6). Following Thulborn’s [59] formula, the allometric gait values ranges between 2.3 to 2.5, indicating a predominantly “trotting” gait. The calculated allometric and morphometric speeds for trackway C are both 3 m/s (S1 Table).

The above described characteristics (i.e., FL > FW, digit III length > digit II and digit IV, and the presence of claw marks) are typical of grallatorid tracks. With an average FL of 13.6 cm, a FL/FW ratio of 1.6 and total divarication of 38°, these C tracks are consistent with the ichnogenus *Grallator*, which is characterized by tulip-shaped tridactyl tracks with a FL < 15 cm, FL/FW ratio of ~ 2, narrow total digit divarication (10–30°), and a digit III that is the longest digit and projects < 50% of the total track length. *Grallator* is commonly attributed to theropod trackmakers (e.g., [59, 79, 113–115]).

## Discussion

### Sedimentology and palaeoenvironment

Massive and horizontally laminated fine-grained sandstone interbeds (Fig 5A, 5B, 5G and 5H) at Highlands that immediately overlie the basalt with a sharp but non-erosional contact indicate an initial rapid deposition of sediments under upper flow regime conditions [116]. A decrease in energy levels is represented by the overlying trough- and planar cross-bedded sandstones (Fig 5E and 5F), which are interpreted as down-current migrating straight- and sinuous-crested aqueous dunes. Laterally, these cross-bedded strata grade into horizontally laminated to massive very fine- to fine-grained sandstones (Fig 5G and 5H) with palaeosurfaces containing asymmetrical and interference ripple marks, a hallmark sedimentary structure of low energy currents during waning floods. The tabular, sheet-like geometry of the sandstones, with thicknesses of < 1.5 m, suggest relatively shallow, unconfined water currents. As attested by the multiple levels of casts of desiccation cracks and invertebrate trails (Fig 5C and 5D), repeated bioturbation and the drying out of the sediment took place before yet another episode of flooding ensued.

The presence of the interference ripples also supports that deposition took place in temporary streams, where these ripple types can form in a two-stage process driven by complex current patterns and bar emergence [117]. The primary ripples form in the low energy currents in downstream flow, however as the flood waters recede further, low water levels result in the partial exposure of in-channel and channel margin bar flanks, which leads to the refraction of downstream currents towards the shoreline. These refracted, much weaker currents produce secondary ripples with a different crest orientation and are preserved amongst the primary ripples.

Overall, the sedimentary facies characteristics of the interbed units at Highlands reflect fluvial deposition within ephemeral streams with initial upper regime flow conditions during flash flooding events (cf. [118, 119]). As flood waters diminished, shallow water deposition in low energy streams and possibly ponds took place after which the sediments were desiccated and buried by ensuing flash flooding event or basaltic lava extrusion. Evidence for aeolian deposits is not preserved in the sandstone interbeds at Highlands.

The sedimentary structures associated with the sandstone interbeds (Fig 5) are evidence for local hiatus in the outpouring of lavas during which sediments were deposited in episodic flash floods. Although the duration of these periods of quiescence in lava outpouring remains unknown without high precision dates, long time gaps with major erosion events that could have removed thick basalt piles are not supported here. This is suggested not only by the non-erosional base of the interbed units (lack of downcutting/incision) but also by the presence of natural sandstone casts that formed over the ropy surface of the underlying pahoehoe basaltic lava flow (Fig 5B). The preservation of the ropy surface, a primary surface feature on basaltic lava flows, is thus an additional evidence for a moderately, and possibly only seasonally, wet climate during the formation of the interbeds, as attested by modern basaltic lava flows in wet tropical climate (e.g., Hawaii), where the revegetation of lava flows and thus alteration of the basalts proceeds rapidly in geological time. The sandstone-filled fissures with clean, sharp contacts that penetrate the basalts (Fig 5A) likely opened up, possibly due to the lava supply dynamics of the pahoehoe feeder tubes (e.g., [120]), prior to the deposition of the sandstone interbeds.

**Fig 13:**
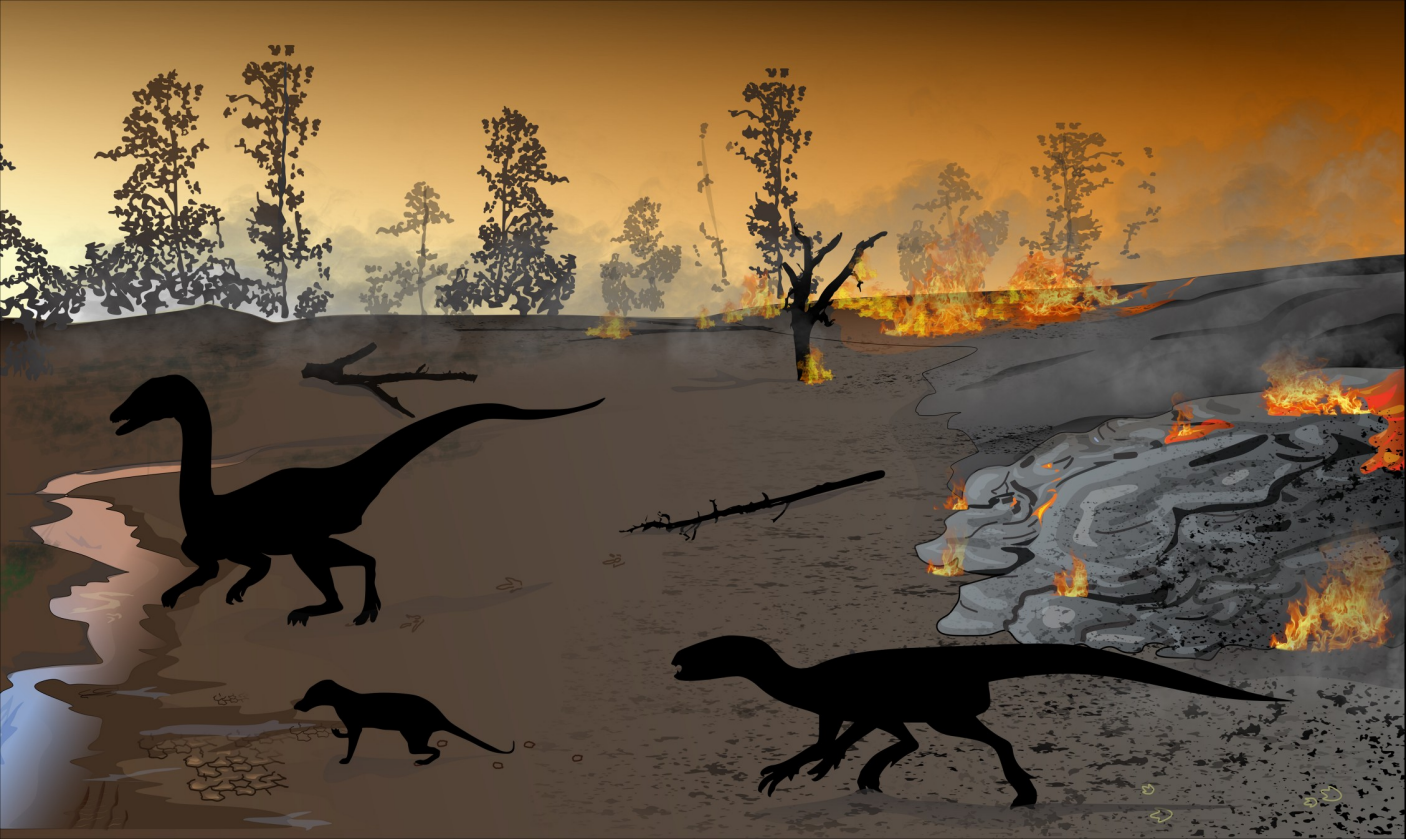
Palaeoenvironmental reconstruction of the Highlands ichnosite at the Pliensbachian–Toarcian boundary. See text for details. Heterodontosaurid silhouette is courtesy of Viktor Radermacher.

In summary, the Highlands palaeoenvironment was characterized by a moderately wet, seasonally dry climate with flash floods that episodically washed over the landscape that had a gentle topography. Possibly, the most significant environmental stressor for the terrestrial biota that inhabited the region, and which included a fairly diverse group of vertebrates (see next section), were the repeated eruptions of basaltic lava flows (Fig 13) and not the aridity of the environment.

### Ichnology

Although relatively diverse, affinity of the vertebrate ichnofauna in the Drakensberg Group is severely understudied [57], and it remains to be seen whether the other vertebrate tracks in the interbeds were made by synapsids, true mammals, protomammals (e.g., [54–56, 58]), or some other reptilian taxa whose fossil remains are unknown to date. In this context, the Highlands discovery is a step in the right direction, because the ichnofauna adds new elements to the tetrapod biodiversity of the Pliensbachian–Toarcian of southern Gondwana and this has ramifications for the global evolutionary trends of vertebrates, in particular ornithischian dinosaurs. In addition, because younger interbeds in central Lesotho seem to be dominated by tracks of presumed mammalian affinity, to date, the Highlands ichnosite is the last known occurrence of diverse dinosaur tracks in the main Karoo Basin. Moreover, because mammalian tracks in the current Mesozoic ichnofaunas are extremely rare [57], it would be significant if the current Pliensbachian–Toarcian ichnological material (especially tracks indicating hopping animals) as well as new findings can be attributed to true mammals, which has a reasonable likelihood given the skeletal evidence of synapsids and true mammals in the upper Elliot and Clarens Formations (e.g., [112]). However, the existing unique ichnofauna and future discoveries in the Lower Jurassic of southern Africa will only realize their optimal biostratigraphic potential if the age of their host sedimentary rocks is resolved with high-precision geochronology.

The pioneer work of Ellenberger is almost 50 years old, and still globally-recognized. However, the post-1980 renaissance in vertebrate ichnology has led to renewed interest in the ichnofaunas of southern Africa and their potential for global correlation. While Ellenberger’s work was not without flaws, some authors (e.g., [121]) perceived ichnotaxonomic errors (over-splitting) where they did not exist: e.g., assuming *Moyenisauropus* a junior synonym of *Anomoepus*, or *Tetrasauropus* a synonym of *Navahopus*. Few other workers have validated these purported synonymies (e.g., [88] in the former case, and [122, 123] in the latter case). Thus, there remain questions about the degree to which various components of the vertebrate ichnofaunas from southern Africa represent endemic rather than pandemic trackmaking species or groups. For example, taken at face value Ellenberger’s extensive ichnofaunal lists suggest a high Early Jurassic diversity, especially among mammaloid trackmakers, relative to other regions. The discovery of a delatorrichnipodid morphotype is suggestive of elements that maybe endemic to the southern hemisphere, the suggestions of Gierliński and Sabath [90] and Gierliński et al. [91, 92] notwithstanding. Ongoing work on the ichnofaunas of the region is generally updating and refining the conclusions of Ellenberger (e.g., [81, 124]), and will help us better understand the ichnotaxonomy, palaeobiology and geological context of the many old and new sites still under investigation. A refined ichnotaxonomy, although not always easy to achieve at the species, genus and family levels, is nevertheless one of the most important keys to an accurate assessment of faunal diversity distribution and questions of palaeogeographic endemism.

The Jurassic palaeogeographic distribution of Delatorrichnopodidae and other ichnofauna in SW Gondwana shows that in spite of the massive outpouring basaltic lavas, which turned the main Karoo Basin into a land of fire (Fig 13) and caused habitat dwindling for the last Karoo vertebrates, life survived this major volcanic event at the end-Pliensbachian. Indeed, the words of the Paul Ellenberger ([54], p. 352) hold true: “Life can only have continued!” [“La vie ne peut qu'avoir continué”] beyond the limits of the main Karoo Basin into the Lower Zambezi Basin and Patagonia.

## Conclusions

Although challenges in understanding the Karoo-Ferrar Large Igneous Province (e.g., sources of the magmas, temporal and spatial relationship of lava flows) remain, this study helps improve the global understating of ecosystem changes associated with the Pliensbachian–Toarcian event by providing new data on the palaeoclimate and palaeoecology in close proximity to the locus of this immense but short-lived magmatic event of global significance. Our study specially demonstrates that the:

1. Track-bearing Pliensbachian–Toarcian sandstone interbeds of the Karoo continental flood basalts at Highlands were deposited in a moderately wet, seasonally dry palaeo-climate that was relatively more humid compared to that of the underlying Clarens Formation, a mostly aeolian succession.
2. Twenty-five vertebrate tracks at Highlands reveal bipedal and quadrupedal trackmakers, and are assigned to a new ichnotaxon, *Afrodelatorrichnus ellenbergeri*, as well as *Brasilichnium*-like and “grallatorid” ichnites.
3. Last inhabitants of the main Karoo Basin were ornithischians, theropods and synapsids, of which small mammaliaforms seem to have persisted for the longest.

This study also demonstrates that the Lower Jurassic continental sedimentary and volcano-sedimentary rock record of southern Africa has a strong potential to provide globally-relevant answers on the triggers of the end-Pliensbachian mass extinction event and the Toarcian biotic turnover (e.g., [20, 46]). Although this extinction event is decisively linked to the giant volcanic events in the Karoo-Ferrar Large Igneous Province, little is known about the background environmental conditions immediately preceding this massive igneous event, whether the environments were progressively stressed or not and what effect the volcanism had on Karoo continental ecology. Without modern equivalents, massive continental volcanic events can only be studied from the geological record, and thus the upper Karoo succession is likely to store answers for these questions and insights, among others, on the underlying causes, nature and timing of the faunal and floral changes at and around the end-Pliensbachian event; and the tempo of the Karoo volcanic eruptive event (i.e., numbers, sizes, volumes of outpouring lava flows), which is directly linked to the rate of volcanic gas addition to the atmosphere, possibly the major trigger for global environmental change.

## Supporting information

S1 Table Ichnological morphometric parameters at Highlands (South Africa), and Chewore (Zimbabwe), including standard track and trackway measurements, and speed calculations. **https://doi.org/10.6084/m9.figshare.9197558.v1**.
(XLSX)
